# Assay interference and off-target liabilities of reported histone acetyltransferase inhibitors

**DOI:** 10.1038/s41467-017-01657-3

**Published:** 2017-11-15

**Authors:** Jayme L. Dahlin, Kathryn M. Nelson, Jessica M. Strasser, Dalia Barsyte-Lovejoy, Magdalena M. Szewczyk, Shawna Organ, Matthew Cuellar, Gurpreet Singh, Jonathan H. Shrimp, Nghi Nguyen, Jordan L. Meier, Cheryl H. Arrowsmith, Peter J. Brown, Jonathan B. Baell, Michael A. Walters

**Affiliations:** 1 Department of Pathology, Brigham and Women’s Hospital, Boston, MA 02115 USA; 2 Institute for Therapeutics Discovery and Development, University of Minnesota, Minneapolis, MN 55414 USA; 3 Structural Genomics Consortium, University of Toronto, Toronto, ON Canada M5G 1L7; 4 Chemical Biology Laboratory, Center for Cancer Research, National Cancer Institute, National Institutes of Health, Frederick, MD 21702 USA; 5 Medicinal Chemistry Theme, Monash Institute of Pharmaceutical Sciences, Monash University, Parkville, VIC 3052 Australia; 6 School of Pharmaceutical Sciences, Nanjing Tech University, Nanjing, 211816 China

## Abstract

Many compounds with potentially reactive chemical motifs and poor physicochemical properties are published as selective modulators of biomolecules without sufficient validation and then propagated in the scientific literature as useful chemical probes. Several histone acetyltransferase (HAT) inhibitors with these liabilities are now routinely used to probe epigenetic pathways. We profile the most commonly used HAT inhibitors and confirm that the majority of them are nonselective interference compounds. Most (15 out of 23, 65%) of the inhibitors are flagged by ALARM NMR, an industry-developed counter-screen for promiscuous compounds. Biochemical counter-screens confirm that most of these compounds are either thiol-reactive or aggregators. Selectivity panels show many of these compounds modulate unrelated targets in vitro, while several also demonstrate nonspecific effects in cell assays. These data demonstrate the usefulness of performing counter-screens for bioassay promiscuity and assay interference, and raise caution about the utility of many widely used, but insufficiently validated, compounds employed in chemical biology.

## Abstract (web summary)

A substantial obstacle in basic research is the use of poorly validated tool compounds with purported useful biological functions. Here, the authors systematically profile widely used histone acetyltransferase inhibitors and find that the majority are nonselective interference compounds.

## Introduction

The majority of primary actives from high-throughput screening (HTS) constitute poorly tractable chemical matter that must be heavily triaged^1,2^. This frequently results from compound-mediated assay interference (i.e., artifacts) or nonselective target modulation, which can originate from several sources, including aggregation, compound light absorbance and/or fluorescence, redox activity, chelation, and nonspecific protein reactivity. In addition to displaying general cytotoxicity, screening compounds can also interfere in certain cell-based assay readouts^3^. The impact of these compounds, including the Pan Assay INterference compoundS (PAINS) of recent notoriety, has been the subject of increased interest and differing views on the utility of structure-based filtering in HTS triage among the medicinal chemistry community^4–7^. But when promiscuous or assay interference compounds (regardless of being flagged as PAINS or not) are not identified or thoroughly characterized, they can be published as real actives in reputable scientific journals leading to their use and propagation in subsequent literature by other researchers^8^. This results in magnification of the number of poor quality biological studies and wasted scientific resources.

Thiol reactivity is a well-known source of assay interference (though aggregation interference is probably more common), as many biologically relevant nucleophiles are thiols, including glutathione (GSH), coenzyme A (CoA), and protein cysteines. Many assay interference and promiscuous bioactive compounds appear to owe their assay promiscuity to nonspecific thiol reactivity, as opposed to the more specific reactivity found in targeted covalent modulators^9^. There are several experimental methods to identify thiol-reactive compounds. Incubating test compounds under assay-like conditions with a thiol-containing reporter such as GSH followed by the detection of thiol-adducts (should they be present) by liquid chromatography–mass spectrometry (LC–MS) is common practice. However, because the most common thiol-based reporters are non-proteinaceous, they may not mimic the local environment found on the surfaces of proteins, and therefore might not recapitulate compound reactivity with physiologically-relevant protein side-chains^10^. At one industrial drug discovery center, a substantial portion of compounds (10/34, 29%) that were unreactive in conventional GSH reactivity assays were flagged as thiol-reactive in ALARM NMR (A LA Assay to detect Reactive Molecules by Nuclear Magnetic Resonance), a protein-based reactivity counter-screen^11^. The authors noted that several of their HTS campaigns were plagued by high rates of ALARM NMR-positive hits, sometimes approaching 50% of screening “hits”.

Chemical probes are now widely used reagents in chemical biology, but their ultimate utility relies on a combination of their potency, selectivity, and mechanism of action. As we and others have previously noted, many commonly used chemical probes suffer from insufficiently characterized mechanisms, poor potencies, and/or numerous off-target effects^12^. That being said, several well-validated epigenetic chemical probes have been reported from different enzymatic classes, including histone deacetylases (HDAC), methyltransferases (HMT), and demethylases^13^. Some of these compounds are in advanced clinical trials or are approved therapeutics. There is, however, a conspicuous lack of such compounds to date for HATs.

HATs have essential roles in gene regulation, nucleosome assembly, DNA repair, and are implicated in certain human cancers^14^. There is considerable interest in HATs as therapeutic targets^15,16^, though there are considerable challenges to targeting this class of epigenetic enzymes^17^. There are several HAT inhibitors reported in the literature^18^, many of which are sold commercially and are featured prominently in epigenetics manuscripts. However, many of these purported tool compounds contain potentially reactive moieties and poor physicochemical properties based on general medicinal chemistry principles, have questionable selectivity for HATs vs. other target classes, or have incomplete structural and purity characterization. Suboptimal chemical screens and follow-up, unknown chemical space preferences, and inherent properties of HATs such as bisubstrate kinetics, active site electrostatic interactions, protein-protein interactions, and complex substrate specificity and regulation have also likely contributed to the paucity of useful HAT probes^9,17,19,20^.

Given the interest in the discovery and development of HAT inhibitors, we systematically characterize reported HAT inhibitor probes. The purpose of this study is two-fold: (1) to assess the potential usefulness of counter-screens such as ALARM NMR for HTS triage and chemical probe validation in an academic setting, and (2) to simultaneously evaluate an entire class of purported tool compounds (i.e., reported HAT inhibitors) for assay interference and potential off-target effects. After performing ALARM NMR, we test reported HAT inhibitors for non-proteinaceous thiol reactivity, in vitro enzymatic activities, aggregation, redox activity, light-based interferences, cellular proliferation, and cellular HAT activity. This undertaking reveals the assay interference and off-target liabilities for an entire class of reported tool compounds (i.e., reported HAT inhibitors), and confirms that the vast majority of these are not reliable biological reagents.

## Results

### Most reported HAT inhibitors are ALARM NMR-positive

We began our studies using ALARM NMR, a protein-based heteronuclear NMR experiment for the identification of thiol-reactive compounds^11,21^. The La antigen contains two cysteine residues, C232 and C245, which are sensitive to thiol-reactive compounds. When covalently modified, these cysteine residues produce characteristic chemical shifts at several nearby ^13^C-labeled leucine residues, namely L249, L294, and L296 (Supplementary Fig. 1). In this assay, compounds are typically tested with and without excess dithiothreitol (DTT), to identify thiol-reactive vs. nonspecific protein perturbation. ALARM NMR and ALARM MS have been employed in the industrial setting to both validate pre-clinical candidates^22^ and to identify nonspecific reactive compounds^23^. We have previously shown that the assay can also flag non-reactive modulators of La antigen conformation; specifically, a class of 4-aroyl-1,5-disubstituted-3-hydroxy-2*H*-pyrrol-2-ones we encountered during an HTS campaign targeting the HAT Rtt109^24^. Compounds with this chemotype did not show evidence of thiol reactivity in several experiments, including an ultra-performance LC (UPLC)-MS GSH adduct assay, but were ALARM NMR-positive independent of DTT, a pattern consistent with nonspecific protein perturbation.

**Fig. 1:**
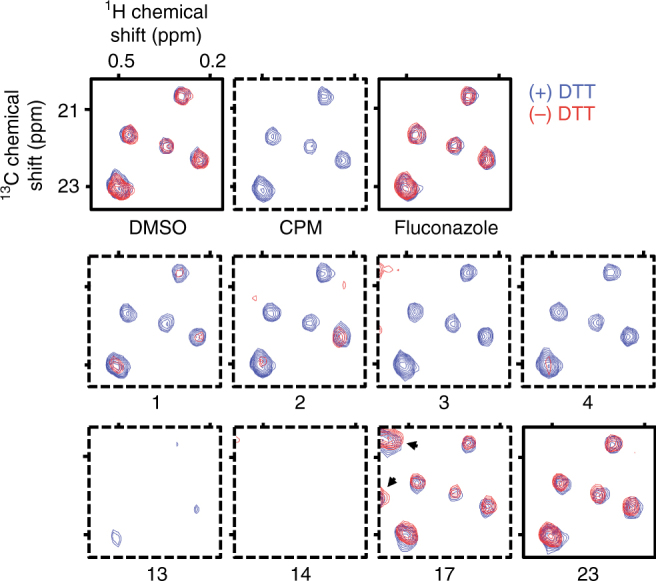
Reported HAT inhibitors perturb the La antigen conformation as assessed by ALARM NMR. Compounds tested at 400 μM final concentrations. Signal intensities (*z*-axis, relative units) normalized to DMSO control. Dashed border, positive readout. CPM (*N*-[4-(7-diethylamino-4-methylcoumarin-3-yl)phenyl]maleimide), positive thiol-reactive compound control; fluconazole, negative thiol-reactive and aggregation compound control. Note treatment of the La antigen with compound **17** results in several new peaks independent of DTT (arrowheads). Data are representative results from one experiment performed with two technical replicates. See Table 1 for additional results and interpretations

Having previously established that ALARM NMR can identify multiple types of assay interference, we tested 23 reported HAT inhibitors, compounds **1**–**23**, by ALARM NMR for evidence of assay interference, which we expected would be predictive of nonspecific target engagement. Remarkably, most of these compounds perturbed the La antigen conformation and were flagged by ALARM NMR (15 out of 23, 65%; Fig. 1 and Table 1).

**Table 1:** Summary of assay interference for reported HAT inhibitors

^a^Common names in parentheses; references for each reported HAT inhibitor are listed in Supplementary Table 1. An expanded data table is also provided (Supplementary Data 1)

^b^(+) DTT, 25 mM DTT present; (−) DTT, 0 mM DTT present; +, positive readout; −, negative readout; /, partial-positive readout; see also Fig. 1; most compounds were tested by two or three independent experiments and yielded similar results

^c^+, adduct detected; −, no adduct detected; I, indeterminate; ND, not determined; see also Methods section for additional interpretation

^d^(*, *), (DTT present, DTT absent); 250 μM test compound concentration; ND, not determined; see also Supplementary Fig. 5

^e^+, detergent-sensitive; −, detergent-insensitive; + (A), detergent-sensitive activation; see also Fig. 2b

Nearly one-half of these reported HAT inhibitors perturbed the La antigen conformation in the absence of DTT (10 out of 23 positive; 2 out of 23 equivocal), a signature of nonspecific thiol reactivity. For example, the alkylidene pyrazolone **1** (C646) has been described as a chemical probe for the mammalian HAT p300^25^. Compound **1** was ALARM NMR-positive in the absence of DTT, consistent with previous reports of nonspecific target engagement and thiol reactivity^26,27^. Other reported HAT inhibitors had ALARM NMR-positive readouts consistent with nonspecific protein perturbation (6 out of 23, 26%) most commonly caused by aggregation. Two such compounds are the natural products **15** (garcinol) and **16** (anacardic acid), both of which have been reported as p300 inhibitors^28,29^.

Not all the reported HAT inhibitors tested were ALARM NMR-positive. The bisubstrate p300 inhibitor **23** (Lys-CoA) had a negative readout, which is expected given its chemical structure^30^. Some compounds were surprisingly ALARM NMR-negative, namely compound **18** (windorphen), which possesses a reactive β-chloro-acrylaldehyde moiety. Its lack of reactivity in ALARM NMR could be caused by stereochemical effects, as only a sample with the *Z*-stereoisomer showed bioactivity in its original report^31^.

### Many HAT inhibitors are thiol-reactive or aggregators

**Fig. 2:**
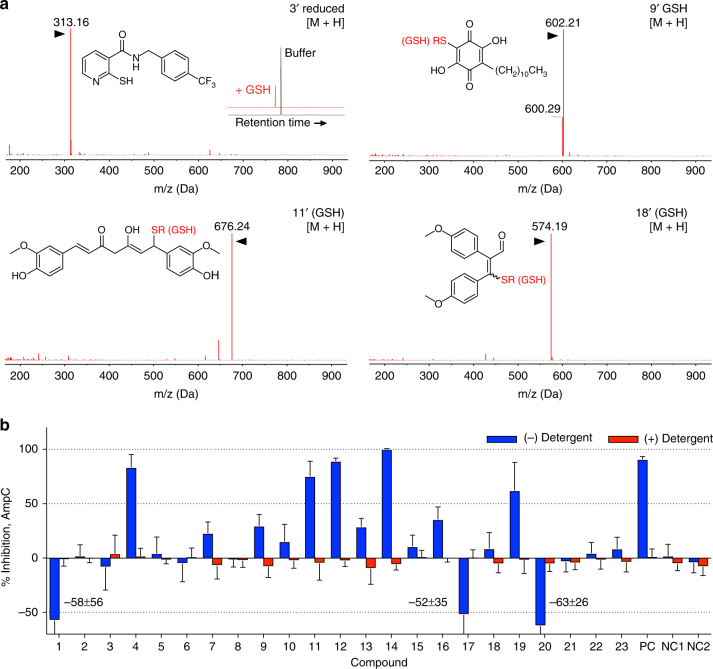
Reported HAT inhibitors exhibit common mechanisms of assay interference. **a** Several reported HAT inhibitors form adducts with GSH (denoted by apostrophe) in vitro as detected by UPLC-MS. Note for compound **3**, the parent compound and the compound detected after treatment with CoA elute at different retention times (see inset). Data are representative results from at least two independent experiments. R, GSH. **b** Several reported HAT inhibitors are positive in an aggregation counter-screen. Compounds were tested for AmpC β-lactamase inhibition ± Triton X-100. Rottlerin, lidocaine, and fluconazole were included as positive control (PC) and negative control (NC1, NC2) compounds, respectively. Data are expressed as mean ± SD pooled from three or four independent experiments each performed with three technical replicates. See Table 1 for additional results and interpretations

To confirm that DTT-dependent ALARM NMR readouts were consistent with thiol reactivity, we tested many of the reported HAT inhibitors for their ability to form covalent adducts with GSH. When incubated with GSH under ALARM NMR-like conditions, several of the DTT-dependent ALARM NMR-positive compounds formed compound-GSH adducts detectable by UPLC-MS (Fig. 2a and Table 1). CoA is another biologically active thiol. As further evidence of nonspecific thiol reactivity, multiple ALARM NMR-positive and GSH-reactive HAT inhibitors also formed adducts with CoA, as detected by UPLC-MS (Supplementary Fig. 2 and Table 1). There was an overall agreement between the ALARM NMR, GSH, and CoA studies with respect to identifying thiol-reactive HAT inhibitors (Table 1).

In addition, gross degradation of parent compound in assay buffer was observed for six of the reported HAT inhibitors during UPLC–MS analyses (Supplementary Data 1). While further characterization of these degradation mechanisms and degradation products is beyond the scope of this work, it highlights the concern that in the absence of purity determination and basic stability studies, the structures of the true biologically active molecules reported in manuscripts featuring these compounds are open to question.

To support that aggregation was responsible for DTT-independent ALARM NMR-positive readouts, compounds **1**–**23** were tested in a standard aggregation counter-screen. In this assay, compounds that form aggregates tend to show strong attenuation of AmpC β-lactamase inhibition in the presence of low concentrations of nonionic detergent^32^. Several of the reported HAT inhibitors (12 out of 23, 52%) showed detergent-sensitive activity, an activity pattern consistent with aggregate formation (Fig. 2b and Table 1). This includes a subset that appears to increase AmpC activity in the absence of detergent. Notably, detergent-sensitive enzymatic activation has been previously described, though the exact mechanism of this phenomenon is unclear^33^. While enzymatic activation has been partially attributed to low-volume assay systems (<10 μL total assay volume)^33^, this is unlikely to explain AmpC activation in our higher-volume system (150 μL total assay volume). With some exceptions, there was general agreement between the ALARM NMR and AmpC counter-screens with respect to flagging HAT inhibitors as aggregators (Table 1).

### Many HAT inhibitors show poor selectivity in vitro

To gauge potential off-target effects in this collection of reported HAT inhibitors, we first performed a pilot selectivity panel on three compounds with either previously reported off-target effects (**1**) and/or substructures with a propensity for nonspecific reactivity (**1**, alkylidene pyrazolone; **2**, disulfide; **3**, isothiazolone)^26,34,35^. These compounds were tested for activity vs. a panel of proteases, kinases, and phosphatases in vitro at concentrations typically used in many published HAT inhibitor experiments (Supplementary Fig. 3). Compound **1** showed > 50% inhibition of several proteases (7 out of 17, 41%), but low levels of inhibition against the tested kinases and phosphatases. By contrast, compounds **2** and **3** showed little activity vs. most of the targets tested. In a more extensive kinase selectivity panel, compound **1** inhibited multiple kinases in vitro, including 26 out of 200 (13%) with >50% inhibition at low micromolar compound concentrations (Supplementary Fig. 4).

**Fig. 3:**
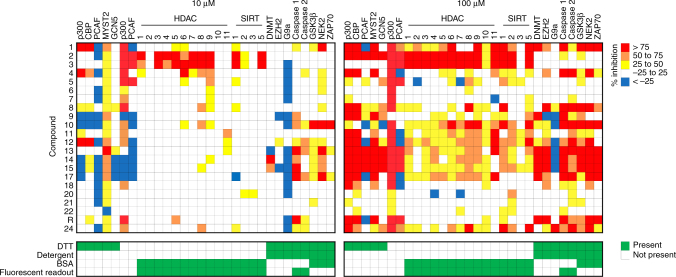
Reported HAT inhibitors modulate multiple targets at concentrations common in biochemical and cell-based assays. Note compounds were tested vs. p300 and PCAF in both the presence and absence of DTT. Bottom panels denote assay condition details. Data are expressed as mean from one experiment performed with two technical replicates. R rottlerin

These initial in vitro results spurred testing of compounds **1**-**23** in a large selectivity panel consisting of HATs, HDACs, sirtuins (SIRTs), HMTs, and several non-epigenetic targets in vitro (Fig. 3). Many of the reported HAT inhibitors identified as thiol-reactive by our aforementioned counter-screens (i.e., **2**, **3, 5**–**11**, **18**) showed minimal activity vs. the five tested HATs at 10 μM when DTT was included in the reaction buffer. A subset of compounds (i.e., **1**, **4**, **12**, **13**) did show potent inhibition of at least one HAT at this concentration when DTT was present. At higher compound concentrations used in some cell-based assays (100 μM), there was a gross trend towards greater HAT inhibition at the expense of HAT selectivity.

To further assess the contribution of thiol reactivity in cell-free HAT inhibition, we also tested the reported HAT inhibitors vs. p300 and PCAF activity in the absence of the reducing agent and electrophile-scavenger DTT (Fig. 3)^36^. For most of the compounds flagged as thiol-reactive by UPLC-MS and ALARM NMR, there was greater inhibition of p300 in the absence of DTT at 10 and 100 μM final compound concentrations (e.g., **1**–**8**). This effect was less pronounced with PCAF, with some notable exceptions (i.e., **2**, **3**, **5**). Interestingly, a subset of compounds appeared to increase PCAF activity in vitro, although the mechanism behind this phenomenon was not further explored.

These activities showed mixed agreement with the original activity reports (Supplementary Table 1), with discrepancies potentially attributable to differences in experimental conditions, particularly scavenging reagent and detergent concentrations. Other factors could include assay technologies, enzyme constructs and concentrations, compound stabilities, and incubation and reaction times.

Many reported HAT inhibitors were active vs. unrelated targets in the biochemical selectivity profiles. At the higher 100 μM compound concentrations, most of the compounds that inhibited multiple HATs also inhibited multiple other non-HAT targets. However, we caution that interpretation of these selectivity data should consider both the experimental conditions and the proposed mechanisms of compound-mediated assay interference. Notably, several assays in this panel included detergent, bovine serum albumin (BSA), and/or DTT, which can mitigate aggregation and thiol reactivity, respectively (Fig. 3, bottom panel). For example, inclusion of DTT and/or BSA may partially explain the decreased on- and off-target activities observed at 10 μM test concentrations. At 100 μM these reagents appear to be less effective in mitigating these types of assay interference, given the aforementioned thiol-reactive and aggregating nature of the majority of compounds **1**–**23**.

### HAT inhibitors exhibit other interference mechanisms

As redox-active compounds represent a significant source of compound-mediated interference in certain biochemical and cell-based assays^37^, we also assessed the majority of compounds **1**–**23** for H_2_O_2_ production in situ. Except for compounds **8** and **16** at relatively high compound concentrations (250 μM), the reported HAT inhibitors did not produce appreciable levels of H_2_O_2_ in situ when assessed by a horseradish peroxidase-phenol red assay (Supplementary Fig. 5 and Table 1)^38^.

Several reported HAT inhibitors were flagged for potential light-based interferences, including absorbance, autofluorescence, and fluorescence quenching (Supplementary Note 1 and Supplementary Figs. 6–8).

### Many HAT inhibitors cause nonspecific effects in cells

**Fig. 4:**
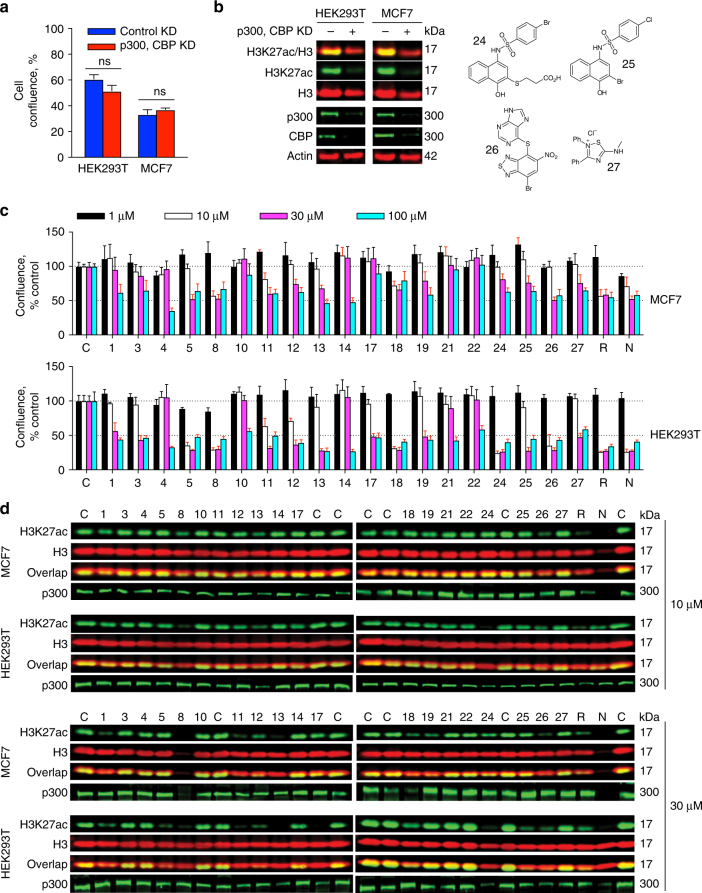
Reported HAT inhibitors cause nonspecific cell assay readouts. **a** Double p300 and CBP knockdowns do not affect cell viability after 2 d. Data are expressed as mean ± SEM of three technical replicates (ns, not significant; two-tailed Student’s *t*-test). **b** Double p300 and CBP knockdowns efficiently reduce H3K27ac levels in HEK293T and MCF7 cells. Molecular weight of probed protein is indicated in kilodaltons (kDa) as verified by molecular weight markers. Data are representative results from one of two independent experiments. **c** Many reported HAT inhibitors exert anti-proliferative effects at low micromolar compound concentrations in (top) MCF7 and (bottom) HEK293T cells as monitored by cell confluence 24 h after addition of compound. Note the nonspecific interference compounds NSC-663284 (N), rottlerin (R), and **24**–**27** (chemical structures shown as inset) demonstrate similar anti-proliferative effects. C, DMSO control. Red error bars, statistically significant difference from respective DMSO controls (*p* < 0.05; two-tailed Student’s *t*-test and Holm–Sidak method). Data are expressed as mean ± SD of three technical replicates and are representative results from one of two independent experiments. **d** Many reported HAT inhibitors also cause nonspecific changes in H3K27ac and p300 levels in cells at low micromolar compound concentrations. Molecular weights of protein analytes are indicated in kDa as verified by molecular weight markers (Supplementary Fig. 9). Note the same known interference compounds from panel **c** can also decrease H3K27ac levels. Data are representative results from one of two independent experiments

Many of the HAT inhibitors we tested have been used not only in biochemical assays, but are also used as tool compounds in cell-based assays. We therefore assessed the effects of reported HAT inhibitors on cell proliferation and on cellular histone acetylation. Double p300/CBP knockdowns did not affect cell viability after 2 days (Fig. 4a), suggesting inhibition of p300/CBP would not be grossly cytotoxic in our experimental system. This knockdown also efficiently reduced levels of p300, CBP, and acetylated histone H3 lysine 27 (H3K27ac) in both HEK293T and MCF7 cells (Fig. 4b). This specific HAT and histone post-translational modification (PTM) were chosen because the majority of the study compounds reportedly target p300/CBP (Table 1)^39^.

Many of the reported HAT inhibitors showed cytotoxic effects in both HEK293T and MCF7 cells at 10 and 30 μM compound concentrations (Fig. 4c). Notably, low-to-mid micromolar compound concentrations are typically used in the original compound reports and in the ensuing literature for HAT inhibitors. In general, compounds were more toxic vs. HEK293T cells. The reported p300 inhibitors **1**, **5**, and **11**–**13** decreased H3K27ac levels in cells, while other reported p300 inhibitors **4**, **14**, and **17**–**19** did not perturb cellular H3K27ac levels (Fig. 4d and Supplementary Fig. 9). In general, reductions in cellular H3K27ac levels were dose-dependent. Several test compounds also decreased cellular p300 levels (Fig. 4d).

In many experimental systems, chemical probes with sufficient specificity and potency would be expected to produce different readouts than nonspecific bioactive compounds when tested at the same concentrations. Several compounds with well-characterized bioassay interferences and promiscuities were therefore tested alongside the reported HAT inhibitors to help gauge the specificity of the cell proliferation and H3K27ac readouts at 10 and 30 μM compound concentrations. Rottlerin and NSC-663284 were used as aggregation and redox-active control compounds, respectively. Compounds **24**–**27**, which are extensively characterized promiscuous bioactive and assay interference compounds, were included as thiol-reactive control compounds^9^. Like many of the reported HAT inhibitors, these interference compounds showed cytotoxic effects and caused decreased H3K27ac levels in both HEK293T and MCF7 cells at 10 and 30 μM compound concentrations (Fig. 4c, d). Decreases in p300 levels were also observed for select interference compounds (Fig. 4d).

Select compounds were further tested to better define the observed compound-mediated effects on cellular proliferation. Compounds **8** and **12** caused dose-dependent increases in caspase 3/7 activity, one hallmark of apoptosis, and also caused dose-dependent increases in membrane permeability, consistent with cytotoxicity (Supplementary Figs. 10 and 11). Like compounds **8** and **12**, the interference compounds NSC-663284, **24**, and **26** increased caspase 3/7 activity and increased membrane permeability (Supplementary Figs. 10 and 11).

We also profiled the effect of reported HAT inhibitors on tubulin acetylation. Notably, tubulin is acetylated by acetyltransferases αTAT1 and MEC-17, and can be deacetylated by HDAC6 and SIRT2^40–43^. Given the nonspecific target engagement profiles of the reported HAT inhibitors and activities vs. various HDACs and SIRTs, cellular tubulin acetylation enzymes may be susceptible to variable target modulation by the reported HAT inhibitors. Overall, those reported HAT inhibitors tested showed variable effects on tubulin acetylation (Supplementary Fig. 12). This observation is perhaps a function of complex dynamics between tubulin acetyltransferases, tubulin deacetylases, and dose-dependent modulation by reported HAT inhibitors.

As certain cellular HAT assays utilize HDAC inhibitors to increase acetylated histone content and control for product depletion by histone deacetylases, we also assessed the effect of reported HAT inhibitors on histone and tubulin acetylation in the presence of suberanilohydroxamic acid (SAHA), a pan-HDAC inhibitor^44^. Compared with cells treated without SAHA, co-treatment of MCF7 cells with SAHA and the reported HAT inhibitors attenuated the decreased H3K27ac levels relative to vehicle controls (Supplementary Fig. 13). These data demonstrate certain reported HAT inhibitors cause decreases in cellular histone acetylation in the absence, but not the presence, of HDAC inhibitors. This observation suggests the benefit of performing cellular HAT assays with and without HDAC inhibitors.

### Concerning literature and cheminformatics trends

**Fig. 5:**
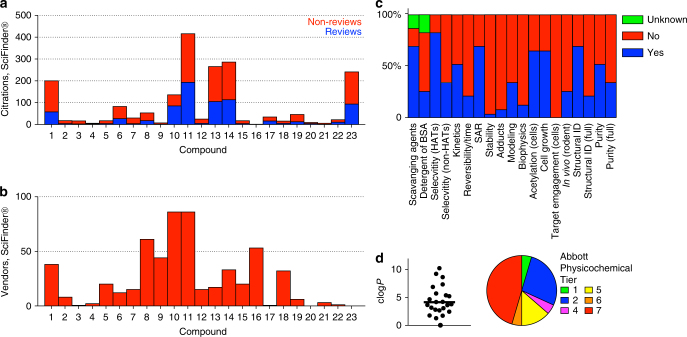
Literature, vendor, and cheminformatics analyses of reported HAT inhibitors show concerning trends. **a** Many HAT inhibitors are highly prevalent in the scientific literature. Shown are the number of reviews (blue subset), non-reviews (red subset), and total unique citations (red plus blue subsets) cataloged in the SciFinder® database for the original reports of compounds **1**–**23** as HAT inhibitors. Data accessed 03 June 2017. **b** Most HAT inhibitors are widely commercially available. Shown are the number of vendors listing compounds **1**–**23** for purchase according to the SciFinder® database, either in-stock or synthesis-on-demand. Data accessed 03 June 2017. **c** Analysis of original publications for compounds **1**–**23** shows common experiments for compound selectivity, mechanism of action, assay interference, target engagement, and purity/identity are not routinely performed. **d** Many of the reported HAT inhibitors tested have poor calculated physicochemical properties (log*P*, APT)

An analysis of the literature shows there is a significant number of scientific publications using and/or citing these reported HAT inhibitors (greater than 1000 total citations, Fig. 5a and Supplementary Data 2). Approximately half of these citations are categorized as reviews according to the SciFinder® search database (Fig. 5a). In this same database, many of these reported HAT inhibitors were available for purchase and/or synthesis-on-demand by multiple vendors (Fig. 5b), indicating many of these compounds are readily accessible to the research community. One note of caution when interpreting these data is that some compounds such as **11** (curcumin) are also marketed for other indications besides HAT inhibition^45^.

An in-depth review of the original reports for compounds **1**–**23** revealed some concerning trends (summarized in Fig. 5c). Many of the original publications failed to include important follow-up experiments, including assays for selectivity, covalent adducts, reversibility, and compound identification and purity. Another trend was the absence of biophysical-based target engagement such as x-ray crystallography, surface plasmon resonance, and thermal shift assays. Molecular modeling was often used to rationalize compound-target engagement and apparent structure-activity relationships (SAR). Many reports described what we characterize as low-quality SAR, featuring either flat activity or apparent SAR that could be best explained by assay interference (SIR, “structure-interference relationships”). Another observation was the use of potentially nonspecific cell-based readouts (i.e., when non-optimized, poorly selective compounds are tested at micromolar compound concentrations) as evidence of on-target engagement, such as cell proliferation and cellular histone acetylation assays.

Since many of these reported HAT inhibitors are non-optimized screening hits, we also determined how compounds **1**–**23** fared against several cheminformatic-flagging tools that are commonly used in HTS triage. These included PAINS, Rapid Elimination Of Swill (REOS), Lilly MedChem Rules, and APT (Abbott Physicochemical Tiering). The first three of these are substructure filters that were designed to help flag compounds that might be promiscuous and/or interference compounds, while the APT is based on physicochemical properties and structural features that have been directly linked to poor solubility, aggregation, and poor cellular permeability^4,46–48^. Therefore, these filters are also useful tools for helping to characterize probe molecules.

Many of the reported HAT inhibitors in our study are flagged by PAINS (11 out of 23, 48%), REOS (14 out of 23, 61%), and Lilly filters (21 out of 23, 91%), and nearly all of the compounds studied herein (22 out of 23, 96%) are flagged by one or more of these tools (Supplementary Data 1). Furthermore, approximately one-third (8 out of 23, 35%) of the compounds tested have unfavorable clog *P* values >5.0, and approximately one-half (11 out of 23, 48%) are categorized in the lowest two tiers by APT (Fig. 5d). Notably, poor calculated physicochemical properties such as high clog*P* and APT values tended to coincide with those compounds identified as aggregators in our profiling study. Prudent and skilled application of these methods, therefore, may alert researchers to helpful counter-screens and estimates about the tractability of these compounds.

## Discussion

One promise of chemical probes is specific and potent target modulation by defined mechanisms^12^. This is critical for studying complex cellular and in vivo processes. The preceding data raise concerns about the selectivity for many compounds used as chemical probes to modulate HAT activity. Greater than half of the compounds tested here showed evidence of nonspecific thiol reactivity, while nearly one-third of the compounds showed assay behaviors consistent with aggregation. Many of these compounds have cell-free potencies in the low-to-mid micromolar ranges, which we have shown to be unreasonable for meaningful target specificity (by comparison, many gold-standard chemical probes have been optimized against their targets to low nanomolar potencies).

This creates a particular challenge for studying HATs, as our data show none of the reported HAT inhibitors contain a substantial number of attributes associated with high-quality chemical probes such as target potency and selectivity, tractable mechanism of target modulation, and meaningful target-based cellular activity^12^. Histone acetylation is a complex biological phenomenon, and we argue the use of suboptimal compounds to probe for HAT cellular functions leads to tenuous, and even false, scientific conclusions that cannot fully account for the contributions of off-target activity, promiscuous reactivity, and/or nonspecific cytotoxicity to cellular, biochemical, or phenotypic readouts.

It is possible that compound-CoA adducts may modulate HAT activity in biochemical and cellular assays. While such a mechanism would be intriguing, it is unlikely to account for the profound assay interference and off-target effects observed in our study. The chemical heterogeneity of the tested HAT inhibitors makes it unlikely such an inhibitory mechanism would apply to every reactive compound in our study. Furthermore, the nonspecific reactivity of the reported HAT inhibitors (as evidenced by reactivity with the La antigen, GSH, CoA, and multiple related and unrelated targets) would be suboptimal for many chemical biology purposes, as multiple adducts and off-target effects could confound the interpretation of cell-based assays. Third, the proposed chemical structures of any compound-CoA adducts in this study appear grossly dissimilar to CoA-based compounds with confirmed binding to HATs^49,50^. In the well-characterized case of the PTC124-AMP adduct inhibiting firefly luciferase, the structure of PTC124 and the enzyme substrate luciferin share significant chemical similarity^51^.

These data, along with previous reports, highlight the utility of performing selectivity testing against multiple related and unrelated targets^52^. We observed even nonspecific compounds did not equally modulate all targets at a given compound concentration (Fig. 3), and among nonspecific electrophiles there can be grossly different selectivity trends (Supplementary Fig. 3). Such trends in protein susceptibility and compound reactivity have been described in various contexts^53,54^. We note target modulation by reactive compounds is dictated by multiple factors, including (but not limited to) the number and microenvironment of protein nucleophiles, the intrinsic function of the biological nucleophile, the assay conditions (including stoichiometry), intrinsic compound electrophilicity, and compound noncovalent binding forces.

This comprehensive evaluation of compound-mediated assay interference demonstrated that some reported HAT inhibitors cause light-based interference including absorbance, autofluorescence, and fluorescence quenching. While fluorescence quenching was observed in the majority of compounds, this occurred at relatively high compound concentrations (low-to-mid micromolar) and high compound:fluorophore ratios. Light-based interference is not overtly problematic if taken into proper account, as these interferences can be corrected with certain experimental designs or circumvented with the use of appropriate orthogonal assays^3,55^. Furthermore, such interference should not be problematic in assays without light-based readouts.

Though not every reported HAT inhibitor was tested, a detailed review of the relevant chemical structures and experimental designs suggests most of these compounds are also likely aggregators or reactive, and nonspecific (Supplementary Note 2).

The cell-based data indicate nonspecific assay interference compounds can disrupt cell proliferation, decrease cellular histone acetylation levels, and even perturb HAT levels. These findings suggest that test compounds have the potential to be misinterpreted as useful HAT inhibitors if the appropriate mechanistic, selectivity, and analytical experiments are not performed. Therefore, histone acetylation can still represent highly useful readouts during the compound discovery and optimization process if confounding factors like assay interference and off-target effects are rigorously considered. However, our data strongly suggest that certain decreases in cellular histone acetylation should be interpreted with caution, particularly at low-to-mid micromolar test concentrations where the causative force(s) behind changes in histone acetylation can be unclear. The decreased perturbations in histone acetylation observed with SAHA co-treatment suggest some utility in performing cell-based experiments with and without nonspecific HDAC inhibitors to more completely assess histone acetylation dynamics. Our data also suggest the potential utility of including known interference compounds as controls in cell-based HAT assays to identify readouts susceptible to assay interference and off-target effects. While our study focused on H3K27ac, it will be interesting to see if other histone acetylation readouts such as H3K9ac, bulk histone acetylation, and even other histone PTMs like histone methylation are susceptible to compound-mediated assay interference. Other cell-based assay systems may also benefit from this practice, especially at early project stages where compound potencies are still in the high nanomolar to low micromolar ranges. The direct comparison of the genetic p300 knockdown and chemical inhibition by reported p300 HAT inhibitors should be interpreted with the caveat that genetic and chemical inhibition of the same target can produce different readouts and phenotypes^56^.

Our literature analysis also reveals some concerning trends. Despite their noted assay interference and off-target liabilities, the original reports of these compounds are widely cited in the scientific literature. While reviews constitute a sizable portion of these citations, we note it can be equally damaging to scientific progress whether a compound is utilized in a primary experiment or the original reports are offered as evidence for scientific conclusions. Our analysis also showed that many of these compounds are commercially available. The ability to readily obtain these compounds is likely one factor contributing to the propagation of these compounds in the scientific literature. A focused analysis of the original HAT inhibitor manuscripts also demonstrates many best-practice experiments to identify and/or mitigate common sources of assay interference and off-target liabilities were not performed.

On the basis of new data presented here and detailed reviews of the original data on these compounds, we do not believe any of the reported small-molecule HAT inhibitors in Table 1 should be recommended as chemical probes in cellular assays. Notably, compound **21**, a reported selective inhibitor of yeast Rtt109^57^, demonstrated a lack of significant assay interference. Though questions remain about its lack of cellular activity, chemical identity, purity, and mechanism of action, additional studies on it or derivatives may eventually support its use. Compound **22**, which also displayed a relatively clean interference profile, is a hit from an AlphaScreen® HTS for MOZ inhibitors and is currently under development^58^. We also note that compound **23** is cell-impermeable. Several peptide derivatives have been reported to overcome this problem, but these were not included as this study focused on small molecules. In light of these results, we recommend that those considering using any of these counter-screen-positive HAT inhibitors critically evaluate the original literature for evidence of potency, selectivity, and useful mechanism of action. As many of the problematic chemotypes encountered in this study are present in other commercially available tool compounds, our results should raise caution about the fitness of other compound classes as well.

There was agreement between the ALARM NMR readout and follow-up counter-screens (Table 1). However, certain compounds may not react with the cysteines on the La antigen reporter protein, but may react with other thiols on different proteins or small molecules like GSH, or vice-versa^11^. Notably, compound **4** was ALARM NMR-positive yet GSH-negative, while compound **18** was GSH-positive yet ALARM NMR-negative. The same observation applies with respect to detergent-sensitive AmpC modulators and ALARM NMR. Several compounds flagged as likely aggregators by the AmpC counter-screen were not identified as such by ALARM NMR (i.e., **1**, **4**, **7**, **19**, and **20**). On the other hand, compound **15** was flagged by ALARM NMR as a potential aggregator, but was negative in the AmpC counter-screen. The reasons for these discrepancies are beyond the scope of this work, but could be explained by individual compound properties (e.g., conformation, reactivity, solubility, aggregation properties, and stability in assay buffer), experimental conditions (e.g., stoichiometry, sensitivity), as well as different overall susceptibilities of GSH, CoA, La antigen, and AmpC to electrophiles and aggregators. For example, compound aggregates can exhibit markedly different binding behaviors as assessed by SPR^59^, while different enzymes can have markedly different susceptibilities to the same aggregator^1^. It follows there is a possibility that compounds may form aggregates in the ALARM NMR and/or AmpC assay(s), but these aggregates fail to grossly perturb protein structure and/or activity. These exceptions should favor the use of multiple counter-screens to de-risk assay interference.

What then should be done with ALARM NMR readouts? First, the data should be interpreted in light of the test compound chemical structure and the therapeutic/scientific context, including fundamental medicinal chemistry principles and a search of the compound “natural history” (i.e., patents, publications, and bioassay results). If ALARM NMR-positive, we generally recommend de-prioritization and potential triage if significant other liabilities are identified. A notable exception could include targeted covalent modulators (e.g., ibrutinib), which can contain weakly reactive warheads incorporated into an optimized, nonreactive scaffold. Such compounds are often designed based on specific mechanistic and therapeutic hypotheses to justify certain risks associated with covalent inhibitor development^60,61^. Depending on factors such as warhead reactivity, reversible binding, and experimental conditions, such compounds may (or may not) be positive by ALARM NMR. In cases of ALARM NMR-positive covalent inhibitors, one may consider performing additional experiments to more extensively gauge target selectivity and reaction mechanisms. In cases with ALARM NMR-negative compounds where assay interference is still suspected, we recommend a combination of additional mechanistic studies and counter-screens for reactivity, aggregation, redox-activity, purity, and stability^9,36^. Given its ability to detect multiple modes of assay interference, ALARM NMR may be a useful first-line tool in HTS triage, helping to direct additional follow-up assays.

The future of HAT inhibitors is not completely bleak. Patent applications have been filed by industry related to cell-active p300-specific inhibitors with encouraging results, including low nanomolar potency in multiple biochemical assays, low nanomolar activity in multiple cell systems, and promising selectivity profiles^62,63^. However, a rigorous and peer reviewed evaluation of this science by other groups is needed for confirmation^69^.

While this report has focused on published HAT inhibitors, our data are likely applicable to other target classes and purported chemical probes. Many problematic chemotypes encountered in this study are also found in other insufficiently validated, purported chemical probes. Our data argue for careful analysis of compound structure and experimental evidence before utilizing tool compounds for chemical biology, and in some cases, even re-examining the validity of compounds with incomplete characterization, despite their wide use and commercial availability.

## Methods

### Compounds and reagents

Reagents were obtained in the highest purity available unless otherwise stated. Most test compounds were purchased as solid powders and used without further purification. Non-commercially available compounds were synthesized in-house using published procedures and characterized for structural identity (NMR, MS) and purity (>95%; UPLC or high-resolution MS). Most manufacturer-provided certificates of analysis reported compound purities verified by elemental analysis and/or purities >95% by HPLC, as well as additional spectroscopic or spectrometric data supporting the assigned compound identity (e.g., NMR, MS). A summary of test compound sources is provided (Supplementary Table 2). Test compounds were typically prepared as 10 mM stock solutions dissolved in neat DMSO and stored at −20 °C under vacuum seals.

### ALARM NMR

The gene encoding amino acids 100–324 of the human La antigen (plus T302N mutation) was cloned into pET28b + vector (Novagen) with both an *N*- and *C*-terminal 6xHis tag (addgene.org, plasmid ID 90209). The sequence identity of the ALARM NMR plasmid construct was verified by Sanger sequencing of the transgene region and next-generation sequencing of the whole plasmid.

Plasmid was transformed into *Escherichia coli* Rosetta cells (Novagen) and cultured in M9 minimal media. The La antigen was labeled by adding ^13^C-labeled amino acid precursors ([3-^13^C]-α-ketobutyrate and [3,3′-^13^C]-α-ketoisovalerate sodium salts; Cambridge Isotope Laboratories; 250 mg L^−1^ final concentrations) to culture medium 30 min before induction with IPTG (1 mM final concentration). Bacteria were collected after incubation at 25 °C for 8 h and washed with ice-cold phosphate-buffered saline (PBS). Washed cells were lysed by French press in ice-cold lysis buffer (50 mM Tris, pH 7.6, 300 mM NaCl, 10% glycerol (v/v), 5 mM β-mercaptoethanol (BME), 5 mM imidazole, 2 mM MgCl_2_, benzonase, and protease inhibitor cocktail), followed by an additional brief sonication step (3 × 15 s pulse sequence) on ice. This lysed solution was centrifuged (30,000×*g* for 30 min, two cycles) to remove cellular debris, then loaded onto a pre-washed Ni-NTA bead column (Qiagen) kept at 4 °C and washed with buffer (50 mM Tris, pH 7.6, 300 mM NaCl, 10% glycerol (v/v), 5 mM BME, 5 mM imidazole). The La antigen was eluted with an elution buffer (50 mM Tris, pH 7.6, 300 mM NaCl, 10% glycerol (v/v), 5 mM BME, imidazole gradient ranging from 5 mM to 0.5 M). The eluted La antigen product was verified by SDS–PAGE to have the correct molecular weight (calculated 32 kDa), and pooled pure fractions were then dialyzed (25 mM sodium phosphate, pH 7.0, 5 mM DTT) in 16 h cycles at 4 °C with gentle stirring, for three total buffer cycles. Aliquots were flash-frozen in liquid N_2_ and stored at −80 °C until further use. Before use, aliquots of ~150 μM protein were incubated in the presence of 20 mM DTT at 37 °C for 1 h, then dialyzed (25 mM sodium phosphate buffer, pH 7.0 (no DTT)) in 16 h cycles at 4 °C with constant nitrogen bubbling and with gentle stirring, for three total buffer cycles.

For standard testing conditions, the [^1^H-^13^C]-HMQC spectra were acquired in 25 mM sodium phosphate buffer, pH 7.0, 10% D_2_O (v/v; CIL) ± 400 μM test compounds delivered from the aforementioned stock solutions, and ± 20 mM non-deuterated DTT. Final concentration of DMSO was 4.0% (v/v). Reaction solutions were mixed, then centrifuged for 5 min (1000×*g*) in non-binding 384-well microplates at RT, then incubated at 37 °C for 1 h and then 30 °C for 15 h before obtaining spectra. Samples were checked for evidence of gross precipitation. Data were recorded at 298 K on a Bruker UltraShield 700 MHz NMR spectrometer equipped with a Bruker 1.7 mm TCI Cryoprobe and Bruker SampleJet autosampler. Samples were tested at 50 μM protein concentrations using 16 scans, 2048 complex points in F2, and 80 points in F1 using standard protein [^1^H-^13^C]-HMQC and water suppression pulse sequences. As further evidence of the correct protein product, the [^1^H-^13^C]-HMQC spectra for the purified La antigen reporter protein matched previously reported La antigen [^1^H-^13^C]-NMR spectra^9,11,21^.

Data were analyzed in TopSpin™ 3.5 (Bruker). Reactions were normalized to equivalent DMSO controls. Non-reactive compounds were identified by the absence of chemical shifts or changes in peak intensities (^13^C-methyl) ± 20 mM DTT. Reactive compounds induced chemical shifts and decreases in peak intensities in certain diagnostic peaks in the absence of DTT. This effect was grossly attenuated when 20 mM DTT was included in an otherwise identical sample. Non-specific perturbations of the La antigen conformation were defined as when significant peak shifts and/or signal attenuation was observed in both the presence and absence of 20 mM DTT in the reaction mixture.

### Selectivity experiments

Selectivity profiles vs. HATs, HDACs, SIRTs, HMTs, proteases, kinases, and phosphatases were performed by Reaction Biology Corporation (Malvern, PA; Fig. 3 and Supplementary Fig. 3). Percent activity was normalized to DMSO-only controls. Reference control compounds were included in each assay.

Relevant reaction procedures for each target class are briefly summarized (see reactionbiology.com for additional details). HATs: reaction buffer 50 mM Tris–HCl, pH 8.0, 50 mM NaCl, 0.1 mM EDTA, ± 1 mM DTT, 1 mM PMSF, 1% DMSO; substrate [^3^H]-acetyl-CoA; reaction time 1 h; end-point measurement. HDACs: reaction buffer 50 mM Tris–HCl, pH 8.0, 137 mM NaCl, 2.7 mM KCl, 1 mM MgCl_2_, 1 mg mL^−1^ BSA, 1% DMSO; fluorogenic substrate p53 residues 379–382 (RHKK(Ac)-AMC) (HDAC1/2/3/6/10), fluorogenic substrate p53 residues 379–382 (RHK(Ac)K(Ac)AMC) (HDAC8); fluorogenic substrate trifluoroacetyl lysine (HDAC4/5/7/9/11); pre-incubation included; reaction time 1–2 h; trichostatin A or TMP269 quencher; fluorescent readout λ_excite_ = 360 nm, λ_emission_ = 460 nm; end-point measurement. SIRTs: reaction buffer 50 mM Tris–HCl, pH 8.0, 137 mM NaCl, 2.7 mM KCl, 1 mM MgCl_2_, 1 mg mL^−1^ BSA, 1% DMSO; fluorogenic substrate p53 residues 379–382 (RHKK(Ac)-AMC) (SIRT1/2/3); fluorogenic substrate Ac-Lys(Succ)-AMC (SIRT5); substrate: 500 µM NAD^+^; reaction time 2 h; nicotinamide quencher; fluorescent readout λ_excite_ = 360 nm, λ_emission_ = 460 nm; end-point measurement (1.5 h development). HMTs: reaction buffer 50 mM Tris–HCl, pH 8.0, 0.01% Brij35, 1 mM EDTA, 1 mM DTT, 1 mM PMSF, 1% DMSO (EZH2); reaction buffer 50 mM Tris–HCl, pH 7.5, 5 mM EDTA, 0.01% Brij35, 5 mM DTT, 0.1 mM PMSF, 5% glycerol, 1% DMSO; 50 mM Tris–HCl, pH 8.5, 5 mM MgCl_2_, 50 mM NaCl, 0.01 Brij35, 1 mM DTT, 1% DMSO (G9a); methyl donor 1 μM *S*-Adenosyl-L-[methyl-^3^H]-methionine; substrate 5 μM histone proteins or 0.05 mg mL^−1^ nucleosomes; pre-incubation time 10–15 min; reaction time 0.5–1 h; end-point measurement. Caspases: reaction buffer 50 mM HEPES, pH 7.4, 1 M citrate, 100 mM NaCl, 0.01% CHAPS, 0.1 mM EDTA, 10 mM DTT; fluorogenic peptide substrate; pre-incubation time 10 min; reaction time 2 h; time-course measurement. Kinases: reaction buffer 20 mM HEPES, pH 7.5, 10 mM MgCl_2_, 1 mM EGTA, 0.02% Brij35, 0.02 mg mL^−1^ BSA, 0.1 mM Na_3_VO_4_, 2 mM DTT, 1% DMSO; substrate [^33^P]-ATP; reaction time 2 h; end-point measurement.

An expanded kinase profile for compound **1** was performed by Cerep/Eurofins (Supplementary Fig. 4). Assays were performed using either homogeneous time-resolved fluorescence or LANCE® time-resolved-FRET formats (see cerep.fr for additional details). In this kinase panel, compound **1** was tested in triplicate at 10 μM final concentrations, and percent activity was normalized to DMSO-only controls. Reference control compounds were included in each assay.

### UPLC-MS assays

Test compounds (1 eq) and reduced L-glutathione (5 eq) were incubated in ALARM NMR buffer (25 mM sodium phosphate, pH 7.0) and 10% DMSO (v/v) at 37 °C for 1 h. Select compounds were incubated up to 24 h for additional adduct analyses. Test compounds were typically incubated at 0.1 mM final concentrations. Samples were injected by an autosampler in 5 μL sample volumes. Samples were analyzed using a Waters UPLC system using a BEH C18 2.1 × 50 mm column. The flow rate was 0.250 mL min^−1^ with a standard gradient starting at 95% Solution A (950 mL H_2_O, 50 mL MeCN, 1 mL formic acid) and ending with 100% Solution B (1000 mL MeCN plus 1 mL formic acid) over 9.0 min. The samples were monitored simultaneously using an evaporative light scattering detector, a diode array detector (214, 220, 244 and 254 nm), and a ZQ mass spectrometer (electrospray positive and negative modes). The CoA adduct assay was performed similarly to the GSH adduct assay, except compounds were incubated with CoA sodium salt (5 eq; Supplementary Fig. 2). Chromatograms and mass spectra were qualitatively analyzed for characteristic compound-GSH/CoA adduct ions based on the chemical structure(s) of proposed adducts.

Gross compound stability was assessed by incubating parent compound in ALARM NMR buffer in the absence of GSH or CoA at 30 °C for 1, 2, and 4 h, and compared to otherwise identical samples containing parent compounds incubated in neat methanol instead of assay buffer. Select compounds were incubated up to 24 h for additional stability analyses. Chromatograms and mass spectra were qualitatively analyzed for the formation of new peaks and/or loss of parent peaks.

### Aggregation assay

Selected compounds were assessed for aggregation using a modified AmpC β-lactamase counter-screen^24,32,64^. Recombinant *E. coli* AmpC (amino acids 20–377) was obtained from MyBioSource.com. Testing was performed in ALARM NMR buffer (25 mM sodium phosphate, pH 7.0) in flat-bottom UV-transparent 96-well microplates in 150 μL reaction volumes. Compounds were tested in triplicate at 10 μM final concentrations in either the presence or absence of freshly-prepared 0.01% Triton X-100 (v/v). Final concentration of DMSO was 2.0% (v/v). Compounds were incubated with ~5 nM AmpC in 147 μL reaction buffer for 5 min at RT, followed by the addition of 3 μL of nitrocefin substrate (100 μM final concentration). Reaction solutions were gently mixed by pipetting up and down three times using a multichannel pipette. Reaction progress was continuously monitored by absorbance at 482 nm for 5 min at RT on a SpectraMax M3 plate reader, and percent activity was calculated from initial reaction rates (*V*_max_). Percent activity was normalized to DMSO-only controls. Fluconazole and lidocaine were included as negative aggregation control compounds. Rottlerin was included as a positive aggregation control compound. Aggregators were defined as compounds with significant detergent-dependent changes in AmpC activity (*t*-test, adjusted *P*-value <0.05; mean differences greater than 30%). Activators were defined as compounds that increased AmpC enzymatic activity.

### Redox activity assay

Selected compounds were assessed for redox activity using a modified horseradish peroxidase-phenol red counter-screen^37,38^. Testing was performed in fresh ALARM NMR buffer (25 mM sodium phosphate, pH 7.0) plus 0.01% Triton X-100 (v/v) in flat-bottom clear polystyrene 384-well microplates in 60 μL reaction volumes. Compounds were tested in triplicate at six final concentrations (240 nM to 250 μM via four-fold dilutions) in either the presence or absence of freshly prepared DTT (1 mM final concentration). Compounds were transferred as 10 mM DMSO stock solutions to microplates via Echo 550 dispenser. Final concentration of DMSO was constant at 2.5% (v/v). Compounds were incubated in 40 μL reaction buffer (with or without DTT) for 15 min, followed by the addition of 20 μL solution containing phenol red and horseradish peroxide (Sigma) dissolved in reaction buffer via ThermoFisher Multidrop dispenser. Final concentrations of phenol red and horseradish peroxide were 280 μM and 60 μg mL^−1^, respectively. The reaction solution was allowed to incubate for 20 min at RT, followed by the addition of 10 μL of 1 M sodium hydroxide via Multidrop dispenser to quench the reaction. After 10 min incubation at RT, H_2_O_2_ production was quantified by measuring absorbance at 610 nm on a SpectraMax M3 plate reader. DMSO and freshly prepared 100 μM H_2_O_2_ were used as negative and positive plate controls, respectively. NSC-663284 and 4-amino-1-naphthol were used as positive redox-active compound controls. Fluconazole was used as a negative compound control. Compounds signals were background-corrected by subtracting DMSO-only plate control signals (absorbance generally between 0.05 and 0.10 at 610 nm). Redox-active compounds were defined as compounds with a mean absorbance >0.2.

### Absorbance and fluorescence spectra

Selected test compounds were assessed for absorption between 300 and 750 nm. Most compound absorption spectra were obtained at 100, 50, and 10 μM final compound concentrations in ALARM NMR buffer (25 mM sodium phosphate, pH 7.0). Final concentration of DMSO was constant at 2.0% (v/v). Compounds were allowed to incubate at RT in buffer for 10 min in UV-transparent polystyrene 384-well plates. Absorbance spectra were then obtained on a SpectraMax M3 or BMG Labtech CLARIOstar microplate reader at 25 °C. Significant absorbance was defined as molar absorptivity constants greater than 2000 M^−1^ cm^−1^ at wavelengths greater than 300 nm.

Selected test compounds were assessed for autofluorescence using an adaption of published procedures^55^. Briefly, fluorophore standards consisted of AlexaFluor 350® (carboxylic acid, Invitrogen), AlexaFluor 488® (carboxylic acid, Invitrogen), AlexaFluor 647® (carboxylic acid, Invitrogen), Texas Red (succinimidyl ester, Invitrogen), FITC (Sigma), 4-methyl umbelliferone (Sigma), resorufin (Sigma), and rhodamine B (Sigma). Test compounds and fluorophore standards were tested in triplicate at six final concentrations (32 nM–100 μM via five-fold dilutions). Final concentration of DMSO was constant at 1.0% (v/v). Compounds and fluorophore standards were transferred as 10 mM DMSO stock solutions to 384-well black polystyrene microplates via Echo 550 dispenser. The plate arrangement was purposefully designed to minimize optical crosstalk by fluorophore standards. All measurements were performed in ALARM NMR buffer (25 mM sodium phosphate, pH 7.0) at 25 °C. Aliquots of 20 μL buffer were dispensed into microplate wells via ThermoFisher Multidrop dispenser. Compounds were shaken for 1 min on a plate shaker, centrifuged briefly for 1 min at 1000×*g*, then allowed to incubate at RT in buffer for 10 min before measuring fluorescence intensity on a SpectraMax M3 plate reader under reduced lighting. Instrument settings: excitation filter wavelength (nm), emission filter wavelength (nm): 4-MU, 340, 450; AlexaFluor 350, 340, 450; FITC, 480, 540; AlexaFluor 488, 480, 540; Rhodamine, 525, 598; Resorufin, 525, 598; Texas Red, 547, 618; and AlexaFluor 647, 570, 671. Bandwidth filters were set to default mode. Fluorescence intensity was measured for each test compound using each of the eight fluorophore standard settings. Fluorophores present on each plate were then used to construct normalized fluorescence dose-responses (“fluorophore-equivalent concentrations”, FEC). Fluorescence intensity for each test compound was converted to the appropriate FEC. Significant autofluorescence was defined as compounds with FECs greater than 100 nM at any compound concentration.

Selected test compounds were assessed for fluorescence quenching using adaptions of previously published procedures^9,24^. Briefly, test compounds and individual fluorophore standards were incubated together. The fluorescence intensity of these mixtures was compared to the fluorescence intensity of the same fluorophore standards minus test compounds. Each microplate contained positive control columns (DMSO plus fixed concentration of a single fluorophore), negative control columns (DMSO), and experimental wells (test compounds plus fixed concentration of a single fluorophore). Test compounds were tested in triplicate at six final concentrations (32 nM to 100 μM via five-fold dilutions) at a fixed 10 μM fluorophore final concentration. Final concentration of DMSO was constant at 2.0% (v/v). Test compounds and fluorophore standards were transferred as 10 mM DMSO stock solutions individually to 384-well Corning black polystyrene microplates (#3677) via Echo 550 acoustic dispenser. All measurements were performed in ALARM NMR buffer (25 mM sodium phosphate, pH 7.0) at 25 °C. Aliquots of 20 μL buffer were dispensed into microplate wells via ThermoFisher Multidrop dispenser. Microplates were shaken for 1 min on a plate shaker, centrifuged briefly for 1 min at 1000×*g*, then allowed to incubate at RT for 10 min before measuring fluorescence intensity on a SpectraMax M3 plate reader under reduced lighting. Instrument settings for each fluorophore were unchanged from the aforementioned autofluorescence studies. Significant fluorescence quenching was defined as signal reduction greater than 25% of the corresponding fluorophore signal at any test compound concentration. BHQ-1 was used as a positive fluorescence quenching control compound^24^.

### Cell lines

HEK293 and MCF7 cells were chosen for cell-based experiments because: (1) several previous reports have tested HAT inhibitors in HEK293 cells^65,66^; and (2) MCF7 cells have a robust response to HDAC inhibitors and generally display less cell death than HEK293 cells in this experimental system.

HEK293T cells were gift from Dr. Sam Benchimol (York University) and MCF7 cells were obtained from ATTC (ATCC® HTB-22™). Cell line identities were verified by STR profiling. *Mycoplasma* contamination was assessed with the MycoAlert™ *Mycoplasma* Detection Kit (Lonza).

### Cell-based assays

Cells were cultured in DMEM supplemented with 10% fetal bovine serum (Winsent), penicillin (100 U mL^−1^), and streptomycin (100 µg mL^−1^). Compounds dissolved in DMSO or matching DMSO-only controls were added to cells for indicated times. Compound effects in cells were determined as described^67^ and are outlined below.

Knockdown experiments employed siRNAs for p300 and CBP, as well as Silencer Select Negative Control No. 2 siRNA (ThermoFisher) that were transfected using RNAiMAX (ThermoFisher) following manufacturer instructions. After 48 h, transfected cells were assayed for toxicity and protein levels as described below.

For western blot analyses, cells were lysed in lysis buffer (20 mM Tris–HCl, pH 8, 150 mM NaCl, 1 mM EDTA, 10 mM MgCl_2_, 0.5% Triton X-100 (v/v), 12.5 U mL^−1^ benzonase (Sigma), complete EDTA-free protease inhibitor cocktail (Roche)). After 3 min incubation at RT, SDS was added to final 1% concentration (w/v). Total cell lysates were resolved in 4–12% Bis-Tris protein gels (Invitrogen) with MOPS buffer (Invitrogen) and transferred for 1.5 h (80 V) onto PVDF membrane (Millipore) in tris-glycine transfer buffer containing 20% methanol and 0.05% SDS. Blots were blocked for 1 h in blocking buffer (5% milk in 0.1% Tween 20/PBS) and incubated with respective primary antibodies: H3K27ac (1:1000, CST #8173), H3 (1:1000, Abcam #10799), p300 (1:2000, Bethyl #A300-358A), CBP (1:1000, Bethyl #A300-362A), actin (1:3000, Abcam #3280), α-tubulin (1:1000, 12G10 DHSB), and α-tubulin-K40ac (1:2000, Abcam #179484) in blocking buffer overnight at 4 °C. After five washes with 0.1% Tween 20/PBS, the blots were incubated with goat anti-rabbit IgG (IRDye 800-conjugated, LiCor #926-32211) and donkey anti-mouse IgG (IRDye 680-conjugated, LiCor #926-68072) antibodies (1:5000) in Odyssey Blocking Buffer (LiCor) for 1 h at RT and washed with 0.1% Tween 20/PBS. The signal was read on an Odyssey scanner (LiCor) at 800 and 700 nm. Representative uncropped images with molecular weight markers are provided (Supplementary Figs. 9, 12, 13).

For cell growth analyses, cells were seeded in 96-well plates, treated with compounds for the indicated times, and monitored over time in a live cell Incucyte™ ZOOM imager (Essen Biosciences). To label apoptotic and dead cells, caspase 3/7 activatable DEVD recognition motif NucView™ 488 DNA intercalating dye (1:1000 dilution, Essen Biosciences) and SYTOX™ green nucleic acid stain (1:1000 dilution, Invitrogen) were used respectively, following manufacturer instructions.

### Cheminformatics

Compound structures were converted to SMILES format and were screened for PAINS, REOS, and Lilly alerts using the FAF-Drugs3 server (http://fafdrugs3.mti.univ-paris-diderot.fr/, accessed 7 Dec 2016)^68^. Abbott Physicochemical Tiering was performed using Pipeline Pilot (Biovia). Log *P* values were calculated using the XlogP3 method.

### Literature analyses

The original reports (including supplementary materials) of compounds **1**–**23** were used for literature analysis (Supplementary Table 1).

For the citation analysis of the original HAT inhibitor manuscripts (Fig. 5a), searching was performed in SciFinder® database (scifinder.cas.org; data accessed 03 June 2017). Duplicate citations were removed by the “remove duplicates” processing tool in the SciFinder® environment and also manual inspection of the downloaded data. Total citations include research articles, reviews, commentaries, patent materials, conference documents, and miscellaneous scientific documents. The total number of reviews was based on the number of designated “General Reviews” using the “document type” analysis tool in the SciFinder® environment. Further analyses were not performed. A file listing each citation in this analysis is provided (Supplementary Data 2).

For the commercial availability analysis (Fig. 5b), searching was performed in SciFinder® database (data accessed 03 June 2017). The total number of commercial sources was based on the “Commercial Sources” analysis tool in the SciFinder® environment. Duplicate vendors were removed by manual inspection of the downloaded data. A file listing each vendor in this analysis is provided (Supplementary Data 2).

For the in-depth review of the original HAT inhibitor manuscripts (Fig. 5c), original sources (including supplementary materials) were evaluated only for the presence or description of each type of experiment; quality was not assessed. “Unknown” values indicate insufficient information provided for evaluation. Full structural and purity identification is based on ACS guidelines.

### Statistical analyses

All graphical data are expressed as mean ± standard deviation (SD) unless stated otherwise. Graphing and statistical analyses were performed using GraphPad Prism 7.0. For aggregation and cell proliferation experiments (Figs. 2b and 4c), statistical significance was evaluated without assuming consistent standard deviation using two-tailed Student’s *t*-test and the Holm-Sidak method to control for multiple comparisons. For siRNA experiments (Fig. 4a), statistical significance was evaluated without assuming consistent standard deviation using two-tailed Student’s *t*-test. Statistical significance was defined as *P*-values <0.05.

### Data availability

All relevant data are available from the authors without restriction. Sequencing data for the ALARM NMR plasmid are available at addgene.org (plasmid ID 90209). Representative ALARM NMR spectra are deposited at Figshare (10.6084/m9.figshare.5401837).

## Electronic supplementary material

Supplementary Information

Descriptions of Additional Supplementary Files

Supplementary Data 1

Supplementary Data 2
