# Supplementary material for: Assay interference and off-target liabilities of reported histone acetyltransferase inhibitors
Source: Nat Commun. 2017 Nov 15;8:1527. doi: 10.1038/s41467-017-01657-3 (PMC5688144; doi:10.1038/s41467-017-01657-3)
Supplement: Supplementary file 1 — Supplementary Information [file 41467_2017_1657_MOESM1_ESM.pdf]

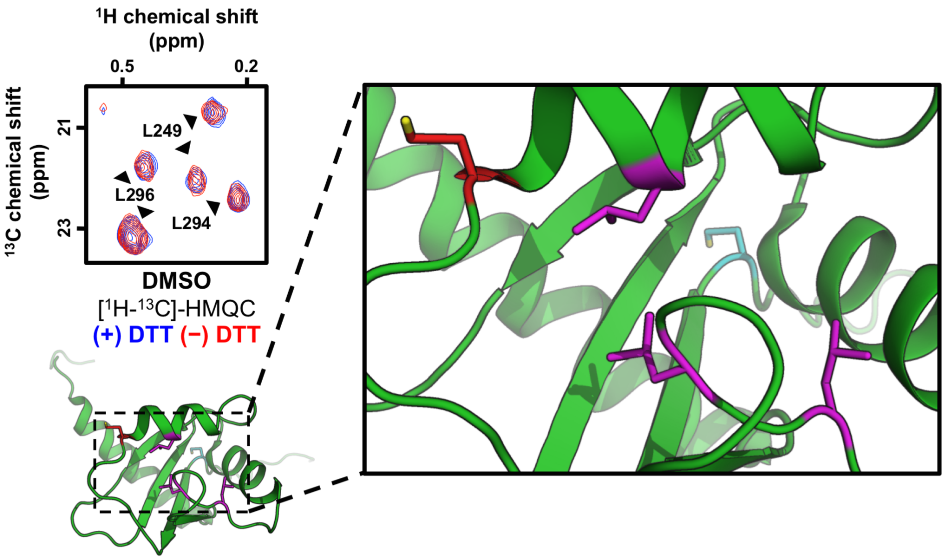

Supplementary Figure 1. Schematic of ALARM NMR counter-screen. The La antigen (green backbone) contains two cysteines, C232 (cyan) and C245 (red), that can react with electrophilic screening compounds, which can consequently perturb the conformation of three nearby leucine residues, L249, L294, and L296 (magenta), as observed by 2D NMR spectroscopy. PDB ID 1OWX[1]. Upper left: representative [^{1}H-^{13}C]-HMQC spectra of the human La antigen showing the chemical shifts for ^{13}C-labelled L249, L294, and L296 in the presence of DMSO. Signal intensities (z-axis, relative units) normalized. Note the chemical shifts and intensities are independent of DTT.

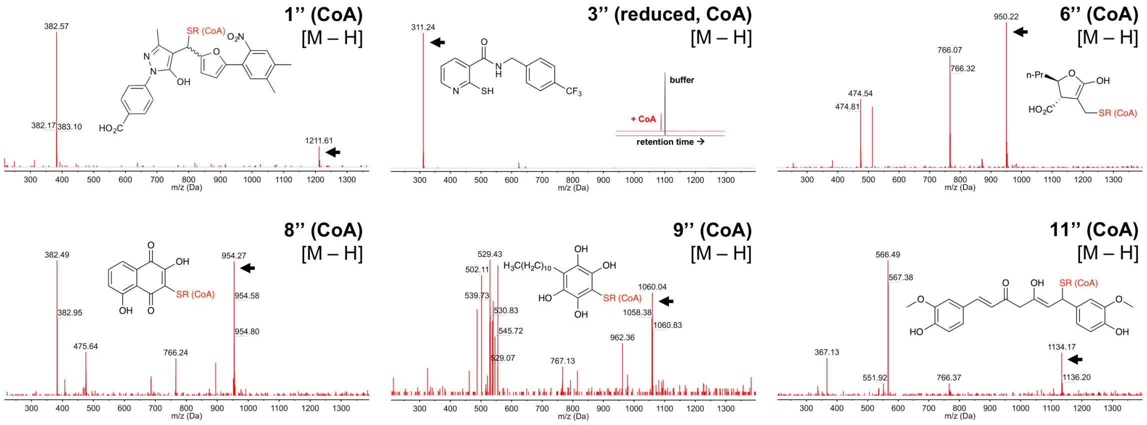

Supplementary Figure 2. Several reported HAT inhibitors form adducts with CoA *in vitro*. Test compounds were incubated in ALARM NMR buffer with excess CoA and analyzed by UPLC-MS. Shown are ESI-negative mode spectra for proposed compound-CoA adducts (denoted by double apostrophe) with arrowheads representing the proposed parent peak. Note for compound **3**, the parent compound and the compound detected after treatment with CoA elute at different retention times (see inset). Insets: proposed chemical structures of compound-CoA adducts. Data are representative results from at least two independent experiments. R, CoA. See **Table 1** for additional results.

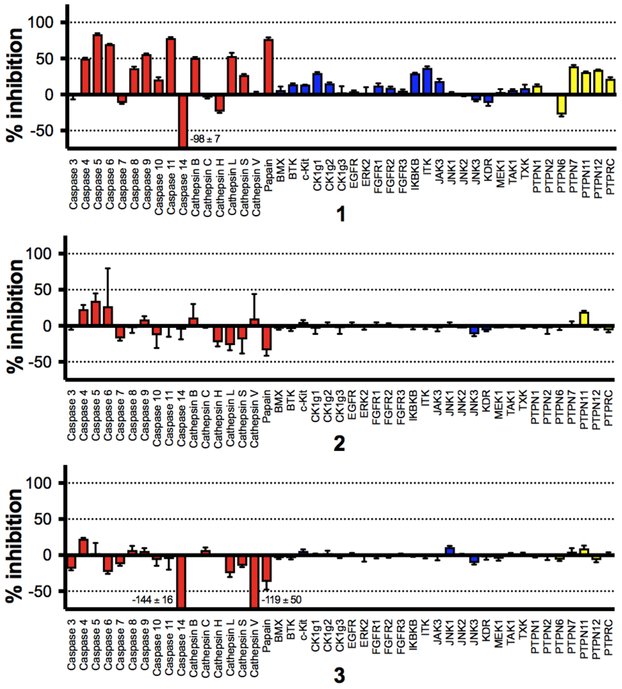

Supplementary Figure 3. Reported HAT inhibitors inhibit multiple targets *in vitro*. Compounds **1-3** were tested for activity modulation versus a panel of kinases, proteases, and phosphatases at 10 μM final compound concentrations. Data are expressed as mean ± SD from one experiment performed with three technical replicates.

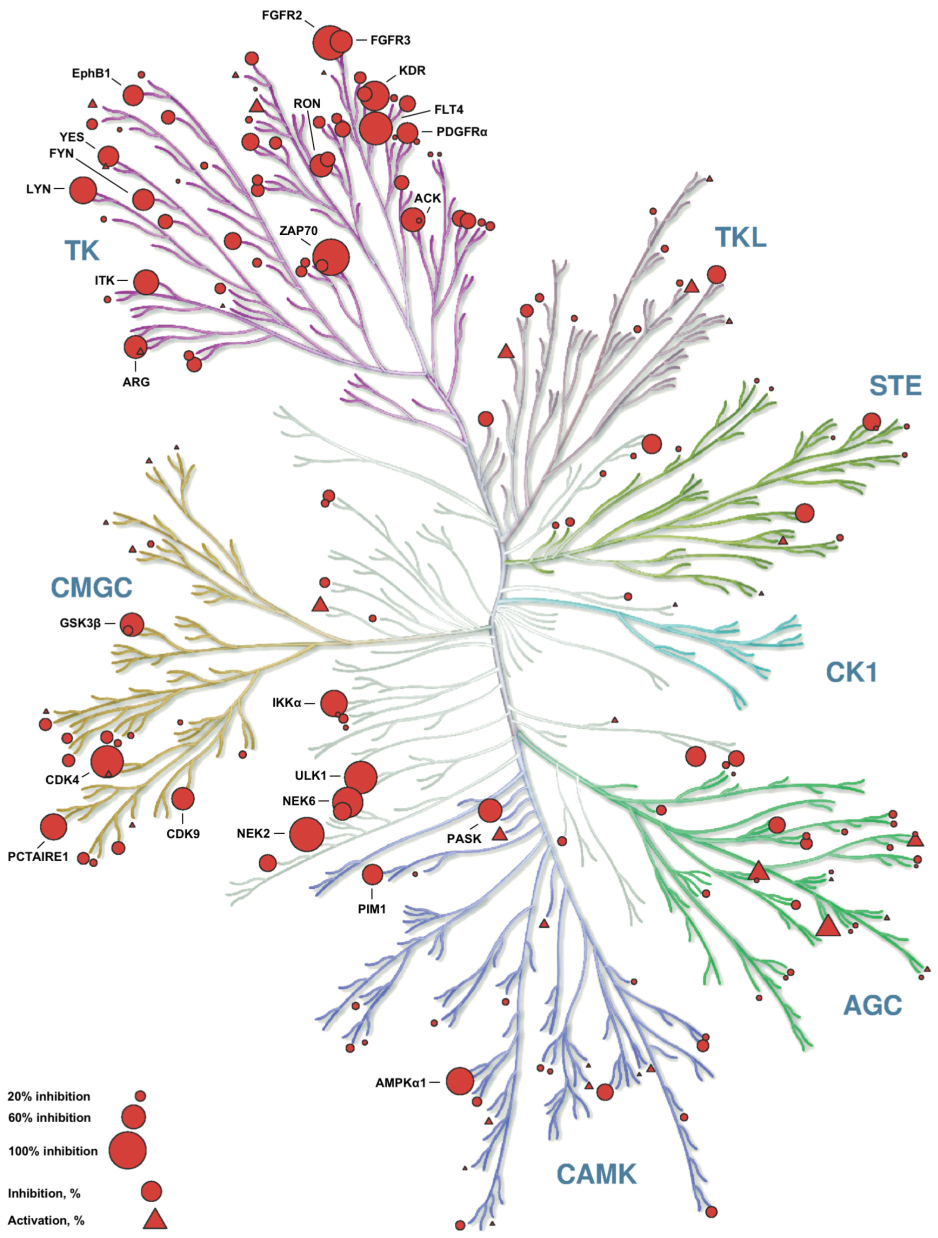

Supplementary Figure 4. Compound 1 inhibits multiple unrelated kinases *in vitro*. Compound **1** was tested for kinase activity modulation at 10 μM final compound concentrations *in vitro***.** The kinase panel consisted of 200 kinases. Data are expressed as mean from one experiment performed with three technical replicates. Kinases with greater than 50% mean inhibition by **1** are individually labelled. Illustration reproduced courtesy of Cell Signaling Technology, Inc. (cellsignal.com)[3,4].

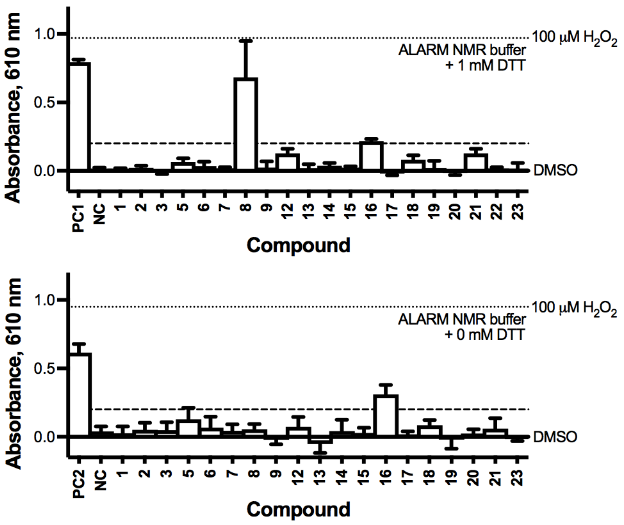

Supplementary Figure 5. Most reported HAT inhibitors are not redox-active. Compounds were tested at 250 μM final concentrations in ALARM NMR assay buffer (25 mM sodium phosphate, pH 7.0; plus 0.01% Triton X-100) using an HRP-PR surrogate assay for H_2O_2 production. Assay was performed with either (top) 1 mM DTT or (bottom) 0 mM DTT in the assay buffer (final concentrations). DMSO and 100 μM H_2O_2, negative and positive plate controls, respectively. NSC-663284 (PC1) and 4-amino-1-naphthol (PC2), redox-active positive control compounds. Fluconazole (NC), negative control compound. Data are expressed as mean ± SD pooled from three independent experiments each performed with three technical replicates. See **Table 1** for summary.

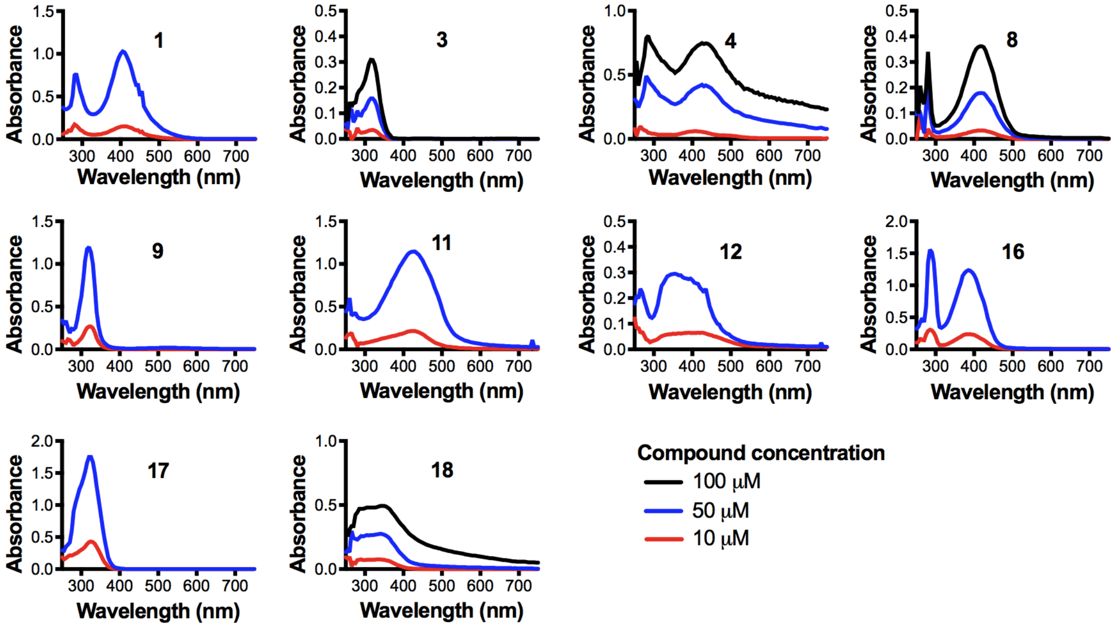

	λ_{max} (nm)	ε_{max} (M^{-1} cm^{-1})		λ_{max} (nm)	ε_{max} (M^{-1} cm^{-1})
**1**	405	18000	**11**	425	20000
**3**	315	2700	**12**	355	5100
**4**	425	6500	**16**	385	22000
**8**	420	3200	**17**	325	30000
**9**	320	21000	**18**	345	4300

Supplementary Figure 6. Several reported HAT inhibitors have potential for absorbance interference. Compound absorbance spectra were obtained at the indicated compound concentrations in ALARM NMR assay buffer (25 mM sodium phosphate, pH 7.0) at room temperature. Data are representative results from two independent experiments.

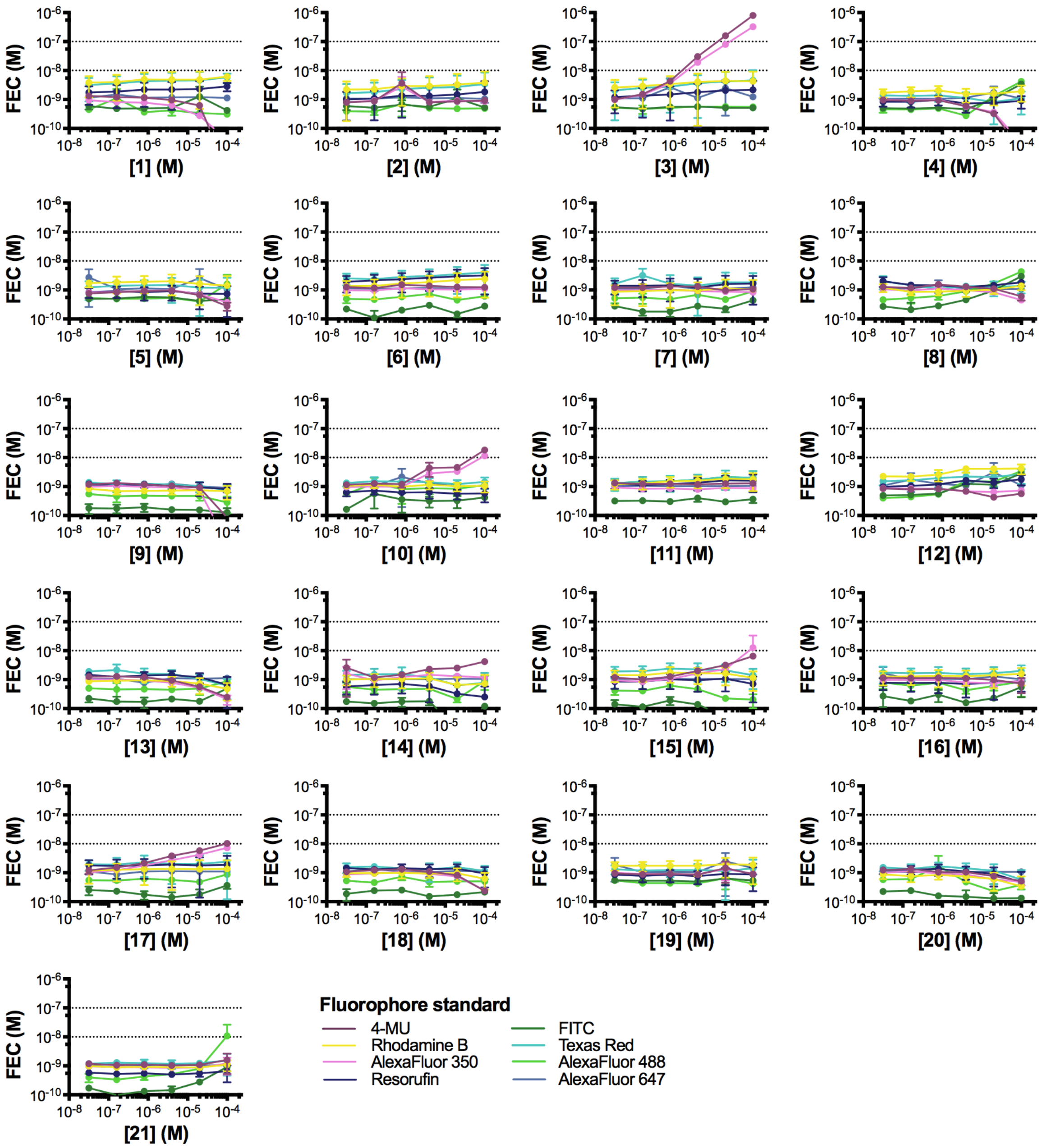

Supplementary Figure 7. Most reported HAT inhibitors do not show significant autofluorescence. Fluorescence intensity of test compounds was measured at the respective fluorophore settings. Compounds were tested in ALARM NMR assay buffer (25 mM sodium phosphate, pH 7.0). Fluorescence signal is expressed in 'fluorophore-equivalent concentrations' (FEC), which are derived from the respective fluorophore standard curves. Data are expressed as mean ± SD from one experiment performed with three technical replicates.

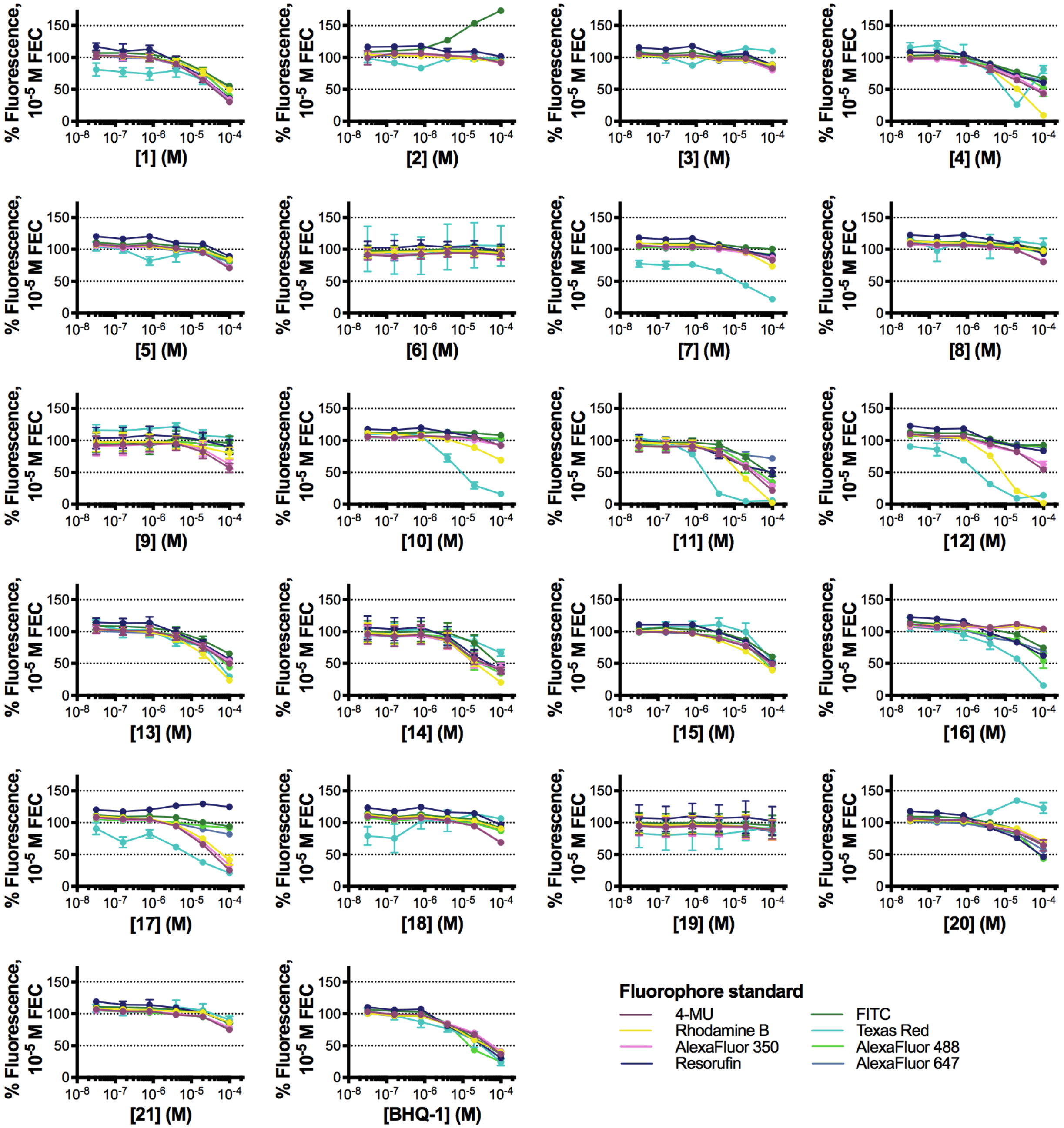

Supplementary Figure 8. Many reported HAT inhibitors exhibit fluorescence quenching behavior. Test compounds were incubated with 10 μM of the respective fluorophore standard, and the fluorescence intensity at the respective fluorophore settings was compared to control. Compounds were tested in ALARM NMR assay buffer (25 mM sodium phosphate, pH 7.0). Fluorescence signal is expressed as FEC. BHQ-1, positive fluorescence quenching control compound. Data are expressed as mean ± SD from one experiment performed with three technical replicates.

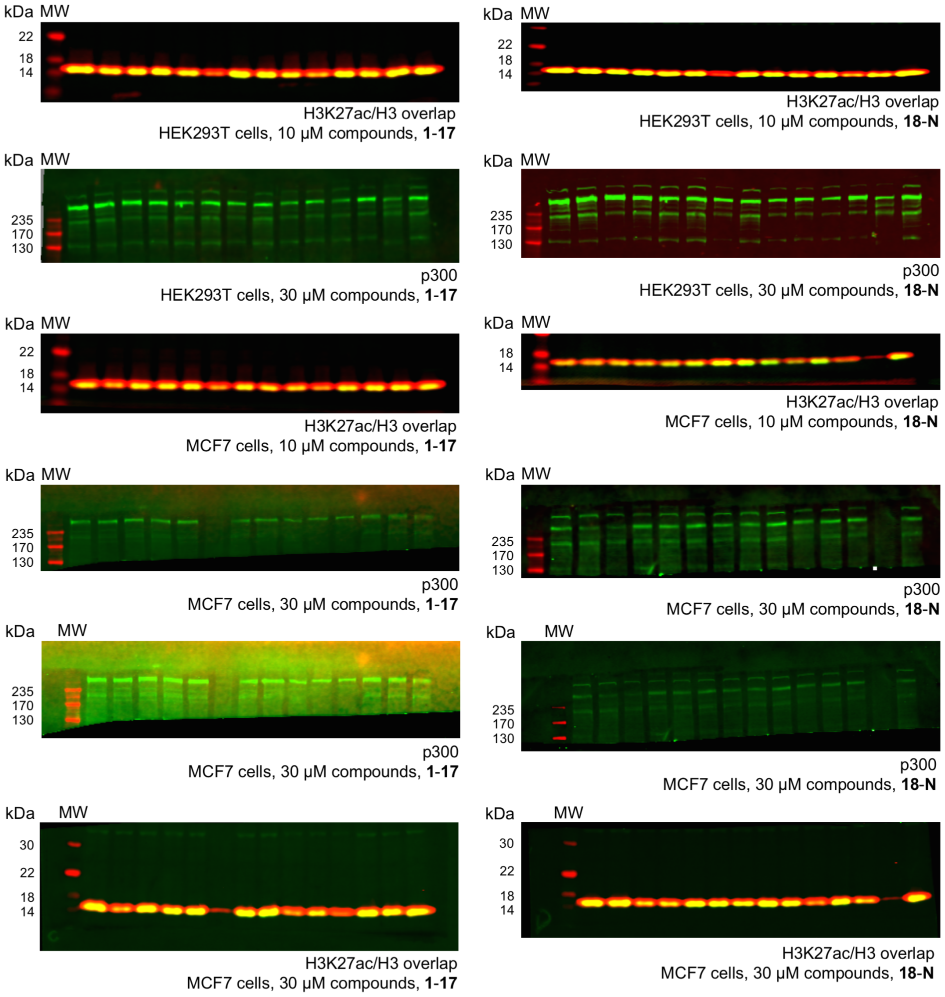

Supplementary Figure 9. Representative gel images. Shown are representative uncropped gels for **Fig. 4D**. Molecular weight markers (MW) are indicated in kDa.

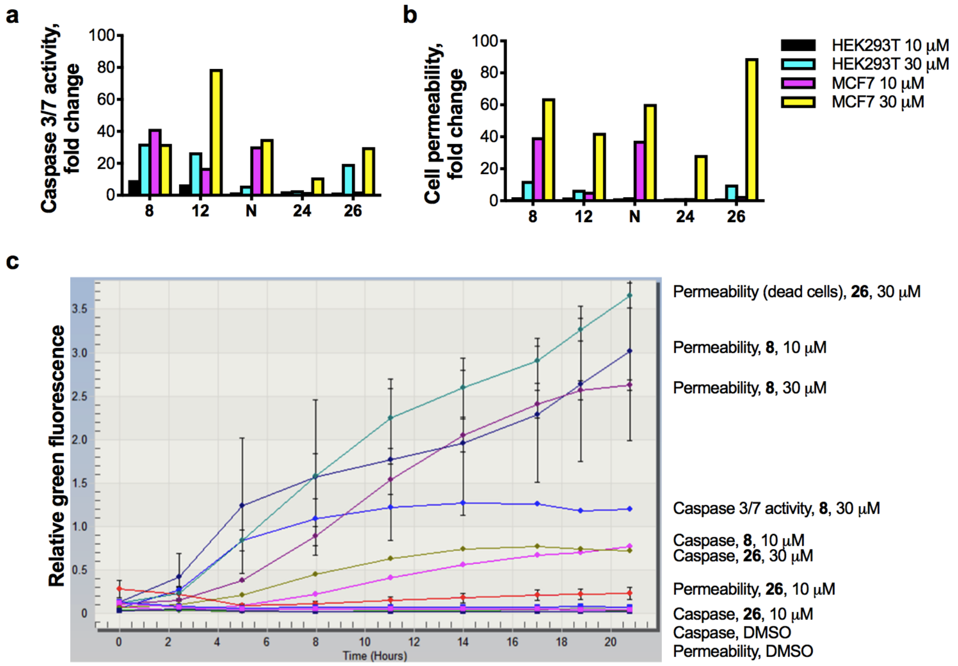

Supplementary Figure 10. Select reported HAT inhibitors induce caspase 3/7 activity and cause cytotoxicity. (a) Select reported HAT inhibitors **8** and **12**, the redox active compound NSC-663284, and the previously characterized nonspecific thiol-reactive compounds **24** and **26** induce caspase activity in HEK293T and MCF7 cells, consistent with apoptosis. Cells were assessed 24 h after compound addition using live-cell caspase 3/7-cleavable fluorescent substrate. Data are from a single experiment. (b) The same compounds increase membrane permeability in HEK293T and MCF7 cells, consistent with cytotoxicity. Cell membrane integrity was assessed by Sytox green dead-cell dye uptake. Only cells with compromised membrane integrity take up the dye that becomes fluorescent inside cells. Data are from a single experiment. (c) Kinetic quantitation of caspase 3/7 activity and cell membrane permeability for compounds **8** and **26** in MCF7 cells. Data are expressed as mean ± SD and are from one experiment performed with three technical replicates.

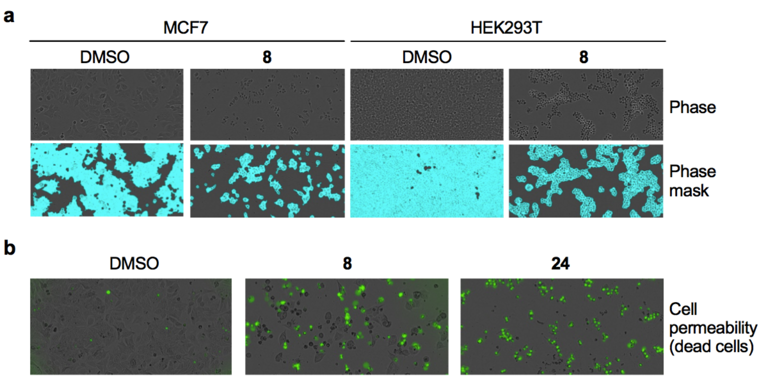

Supplementary Figure 11. Reported HAT inhibitors are cytotoxic and induce apoptosis at micromolar compound concentrations. Shown are representative images of cell confluence, apoptosis, and cell permeability data reported in **Fig. 4** and **Supplementary Fig. 10** for cells treated with 30 μM compound, final concentration. (a) The reported HAT inhibitor compound **8** causes cell death in MCF7 and HEK293T cells as shown by cell confluence studies. Data are from a single experiment. (b) Compound **8** and **24** (a previously characterized nonspecific thiol-reactive compound) are cytotoxic in MCF7 cells as determined by membrane integrity studies. Data are from a single experiment.

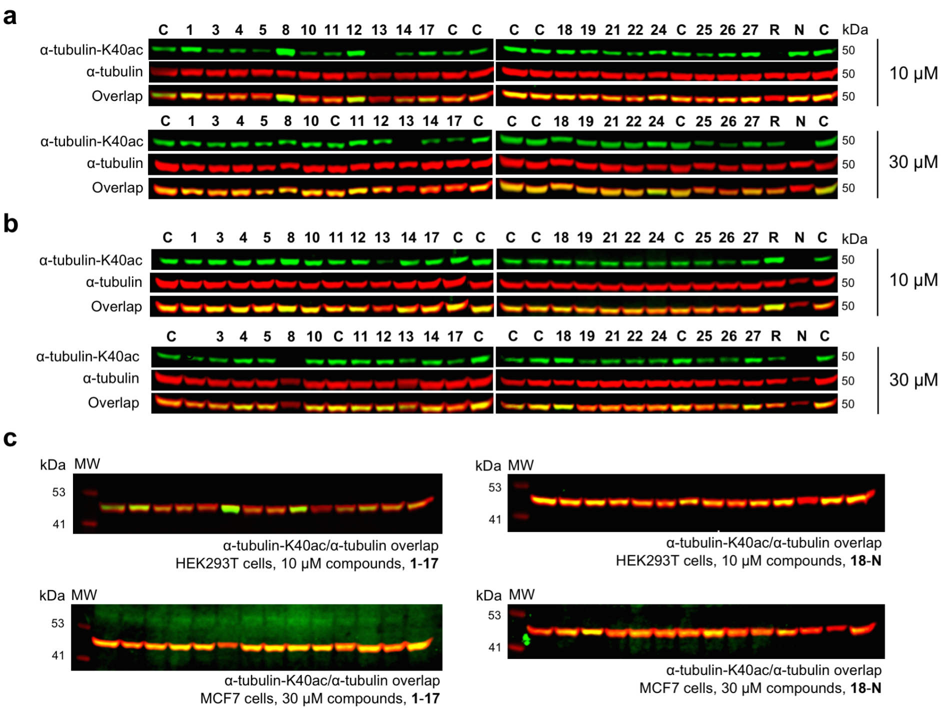

Supplementary Figure 12. Reported HAT inhibitors show variable effects on tubulin acetylation in HEK293T and MCF7 cells. Western blots of α-tubulin and α-tubulin-K40ac in (a) HEK293T and (b) MCF7 cells treated with 10 or 30 μM test compounds final concentration. Western blots were normalized to α-tubulin. C, DMSO control; N, NSC-663284; R, Rottlerin. Data are from a single experiment. Molecular weights of protein analytes are indicated in kDa as verified by molecular weight markers (MW). (c) Representative uncropped gel images from panels (a) and (b). MW are indicated in kDa.

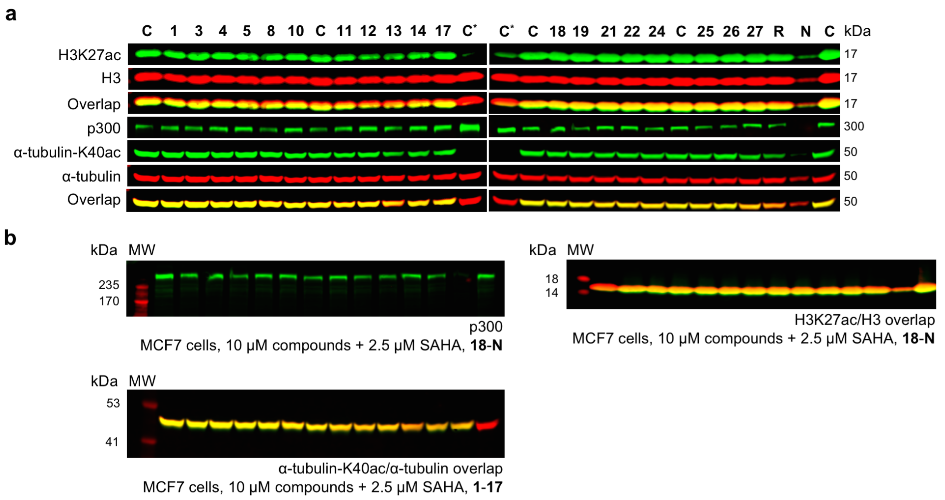

Supplementary Figure 13. Reported HAT inhibitors do not significantly perturb H3K27 and tubulin acetylation in MCF7 cells co-treated with 2.5 μM SAHA. (a) Western blots of H3K27ac, H3K27, actin, p300, α-tubulin and α-tubulin-K40ac in MCF7 cells treated for 24 h with 10 μM test compounds final concentration. Note: compare to **Fig. 4** for analogous non-SAHA experiment. Western blots for H3K27ac and α-tubulin-K40ac were normalized to actin and α-tubulin, respectively. C, DMSO control; C*, DMSO control with no SAHA; N, NSC-663284; R, Rottlerin. Data are from a single experiment. Molecular weights of protein analytes are indicated in kDa as verified by molecular weight markers (MW). (b) Representative uncropped gel images from panel (a). MW are indicated in kDa.

Supplementary Table 1. Original references and associated target activities for reported HAT inhibitors.

		IC$_{50}$ (μM)						
**Cpd**	**Reference**	**p300/CBP**	**GCN5**	**PCAF**	**Tip60**	**Other HATs**	**Trapping/ reducing agents**	**Aggregation mitigation (detergent, decoy protein)**
**1**	[5]	1.6	< 10% inhibition @ 10 μM	< 10% inhibition @ 10 μM		SAS (< 10% inhibition @ 10 μM); MOZ (< 10% inhibition @ 10 μM); Rtt109 (< 10% inhibition @ 10 μM)	1-5 mM DTT	40-50 ng/μL BSA; +/- 0.01% v/v Triton X-100
**2**	[6]	59	> 100	33	~2		None	None
**3**	[7]			130 (H3 peptide substrate); 35.5 (H4 peptide substrate)			None	0.1% w/v BSA; 0.8% v/v Triton X-100
**4**	[8]	p300 (2.9); CBP (1.1)	"practically inactive"	"practically inactive"			1 mM DTT	None
**5**	[9]	p300 (1.98, fluorescence); p300 (128, HotSpot); CBP (32% inhibition @ 100 μM)	33.9 (HotSpot)	34.7 (HotSpot)	2% inhibition @ 100 μM	MYST2 (1% inhibition @ 100 μM, HotSpot); MYST4 (-28% inhibition @ 100 μM, HotSpot)	1 mM DTT	None
**6**	[10]	CBP (500)	100				Unknown	Unknown
**7**	[11]		~500				1 mM DTT	None
**8**	[12]	25		~50% inhibition @ 50 μM			Unknown	Unknown
**9**	[13]	25% inhibition @ 25 μM		7.2	25% inhibition @ 25 μM		Unknown	Unknown
**10**	[14]	p300 (30); CBP (50)		60	70		None	Unknown
**11**	[15]	p300 (~25); CBP (~25)		< 10% inhibition @ 100 μM			None	None
**12**	[16]	5	30% inhibition @ 5 μM	< 1% inhibition @ 5 μM			1 mM DTT	None
**13**	[17]	5		7			1 mM DTT	None
**14**	[18]	8.5		5			1 mM DTT	None
**15**	[19]	30% inhibition @ 200 μM		3% inhibition @ 200 μM	74	MOF (47)	1 mM DTT	0.01-0.1% v/v Triton X-100; 0-50 ng/uL BSA
**16**	[20]	p300 (~25); CBP (~25)		~50	< 10% inhibition @ 100 μM		1 mM DTT	None
**17**	[21]		40% inhibition @ 40 μM				2 mM DTT	0.05% v/v Triton X-100
**18**	[22]	p300 (4.2); CBP (51.3)	> 100	> 100		KAT5 (> 100); MYST2	1 mM DTT	None

						(> 100); MYST4 (> 100)		
**19**	[23]	74% inhibition @ 50 μM	< -25% inhibition @ 50 μM	< -25% inhibition @ 50 μM			1 mM DTT	None
**20**	[24]				~500 μM		1 mM DTT	None
**21**	[25]	< 25% inhibition @ 100 μM	< 25% inhibition @ 100 μM			Rtt109 (0.056)	0 or 1 mM DTT depending on assay	0.0005% v/v Pluronic F-68 0.01% or v/v Triton X-100 depending on assay; 0.1 mg/mL BSA or 0.01% NEM-treated BSA depending on assay
**22**	[26]			> 100		MOZ (0.38 μM K_d); MOZ (2.4 μM K_i); MOF (> 100 μM)	1 mM DTT	0.01% v/v Tween-20; 0.02% m/w chicken egg white albumin
**23**	[27]	0.5		200			1 mM DTT	None

Supplementary Table 2. Compound sources and miscellaneous notes.

Name	Vendor	Catalog #	Comments
Alexa Fluor 350	ThermoFisher	A33076	Carboxylic acid; protect from light
Alexa Fluor 488	ThermoFisher	A33077	Carboxylic acid; protect from light
Alexa Fluor 647	ThermoFisher	A33084	Carboxylic acid; protect from light
4-Amino-1-naphthol	TCI America	A0366	HCl salt
BHQ-1	Biosearch Technologies	BNS-5051N	Amidite form
BSA	Sigma-Aldrich	05470	Bovine serum albumin; lyophilized powder; crystallized; prepare fresh and dissolve in ALARM NMR buffer
CPM	Sigma-Aldrich	C1484	*N*-[4-(7-diethylamino-4-methylcoumarin-3-yl)phenyl]maleimide; protect from light
DTT	Sigma-Aldrich	D5545	DL-Dithiothreitol; prepare stock solution fresh for each experiment (poor aqueous half-life)
FITC	Sigma-Aldrich	F7250	Fluorescein isothiocyanate; protect from light
Fluconazole	Sigma-Aldrich	F8929	
H_2O_2	Sigma-Aldrich	216763	Hydrogen peroxide; contains inhibitor, 30 wt. % in H_2O; prepare stock solution fresh for each experiment
4-Methyl umbelliferone	Sigma-Aldrich	M1381	Protect from light
NSC-663284	Sigma-Aldrich	N7537	
Resorufin	Sigma-Aldrich	424455	Protect from light
Rhodamine B	Sigma-Aldrich	79754	Protect from light
SAHA	Alexis Biochemicals		Suberoylanilide hydroxamic acid
Texas Red	Thermo Fisher	T20175	Succinimidyl ester; protect from light
Triton X-100	Sigma-Aldrich	T9284	Prepare 10% solution (v/v) fresh for each experiment (can produce H_2O_2)
**1**	Sigma-Aldrich	SML0002	C646; protect from light
**2**	Santa Cruz Biotechnology	sc-397052	NU-9056
**3**	–	–	PU141; synthesized in-house (NN, JBB) as previously described[7]
**4**	XcessBio	M60257	EML425; protect from light
**5**	Tocris Bioscience	5045	L002
**6**	Sigma-Aldrich	M2449	MB-3
**7**	Sigma-Aldrich	C9873	CPTH2
**8**	Tocris Bioscience	4761	Plumbagin
**9**	Sigma-Aldrich	E1406	Embelin
**10**	Sigma-Aldrich	E4143	(−)-Epigallocatechin gallate
**11**	Santa Cruz Biotechnology	sc-200509	Curcumin; protect from light
**12**	Santa Cruz Biotechnology	sc-397036	HAT Inhibitor II; protect from light
**13**	Santa Cruz Biotechnology	sc-200891	Garcinol; protect from light
**14**	Sigma-Aldrich	A7236	Anacardic acid

**15**	Selleck Chemicals	S7476	MG149
**16**	Cayman Chemical	14482	Gossypol
**17**	EMD Millipore	382115	CTK7A
**18**	Sigma-Aldrich	SML0899	Windorphen
**19**	Key Organics US	HG-0032	LoCAM
**20**	Axon	2339	TH1834; HCl salt
**21**	Enamine	Z14250979	*N*-[(2-chloro-6-fluorophenyl)methyl]-2-(2,5-dioxo-4-phenyl-4-propylimidazolidin-1-yl)-*N*-methylacetamide
**22**	–	–	CTx-1; synthesized in-house (NN, JBB; forthcoming manuscript)
**23**	–	–	Lys-CoA; synthesized in-house (JHS, JLM) as previously described[28]
**24**	eMolecules	8646152	Thiol reactive, redox-active; **6a** from previous study[2]
**25**	eMolecules	1165100	Thiol reactive, redox-active; **6e** from previous study[2]
**26**	eMolecules	6447334	Thiol reactive; **2a** from previous study[2]
**27**	eMolecules	1947931	Thiol reactive; **3a** from previous study[2]

Supplementary Note 1. Light-based interference of reported HAT inhibitors. We assessed the reported HAT inhibitors for absorbance and fluorescence interference, as many HTS and follow-up assays (both biochemical and cell-based) will either quantify a light-based signal or utilize microscopy for phenotypic studies. Overall, 10/22 (45%) of the reported HAT inhibitors tested were flagged for potential absorbance interference between 300 and 425 nm wavelengths (**Supplementary Fig. 6**). Few reported HAT inhibitors showed significant levels of autofluorescence when tested at eight common fluorophore wavelengths (**Supplementary Fig. 7**). By contrast, the majority of the reported HAT inhibitors tested (16/21, 76%) showed evidence of fluorescence quenching by at least one fluorophore at low-to-mid micromolar compound concentrations (**Supplementary Fig. 8**).

Supplementary Note 2. Untested reported HAT inhibitors. A literature search identified multiple other compounds reported as HAT inhibitors that were not tested in this report[29-46]. Based on the chemical structure and review of the original manuscripts, many of these compounds show weak potency, and many are also likely thiol-reactive, aggregators, and likely nonspecific in their target engagement under common HAT assay conditions. Other, non-drug-like natural products would benefit from additional characterization[47].

Supplementary References

1. Jacks, A. et al. Structure of the C-terminal domain of human la protein reveals a novel RNA recognition motif coupled to a helical nuclear retention element. *Structure* **11**, 833-843 (2003).

2. Dahlin, J.L. et al. PAINS in the assay: chemical mechanisms of assay interference and promiscuous enzymatic inhibition observed during a sulfhydryl-scavenging HTS. *J. Med. Chem.* **58**, 2091-2113 (2015).

3. Chartier, M., Chenard, T., Barker, J. & Najmanovich, R. Kinome Render: a stand-alone and web-accessible tool to annotate the human protein kinome tree. *PeerJ* **1**, e126 (2013).

4. Manning, G., Whyte, D., Martinez, R., Hunter, T. & Sudarsanam, S. The protein kinase complement of the human genome. *Science* **298**, 1912-1934 (2002).

5. Bowers, E.M. et al. Virtual ligand screening of the p300/CBP histone acetyltransferase: identification of a selective small molecule inhibitor. *Chem. Biol.* **17**, 471-482 (2010).

6. Coffey, K. et al. Characterisation of a Tip60 specific inhibitor, NU9056, in prostate cancer. *PLOS ONE* **7**, e45539 (2012).

7. Furdas, S.D. et al. Synthesis and biological testing of novel pyridoisothiazolones as histone acetyltransferase inhibitors. *Bioorg. Med. Chem.* **19**, 3678-3689 (2011).

8. Milite, C. et al. A novel cell-permeable, selective, and noncompetitive inhibitor of KAT3 histone acetyltransferases from a combined molecular pruning/classical isosterism approach. *J. Med. Chem.* **58**, 2779-2798 (2015).

9. Yang, H. et al. Small-molecule inhibitors of acetyltransferase p300 identified by high-throughput screening are potent anticancer agents. *Mol. Cancer. Ther.* **12**, 610-620 (2013).

10. Biel, M., Kretsovali, A., Karatzali, E., Papamatheakis, J. & Giannis, A. Design, synthesis and biological evaluation of a small molecule inhibitor of the histone acetyltransferase Gcn5. *Angew. Chem. Int. Ed.* **43**, 3974-3976 (2004).

11. Chimenti, F. et al. A novel histone acetyltransferase inhibitor modulating Gcn5 network: cyclopentylidene-[4-(4'-chlorophenyl)thiazol-2-yl)hydrazone. *J. Med. Chem.* **52**, 530-536 (2009).

12. Ravindra, K. et al. Inhibition of lysine acetyltransferase KAT3B/p300 activity by a naturally occurring hydroxynaphthoquinone, plumbagin. *J. Biol. Chem.* **284**, 24453-24464 (2009).

13. Modak, R. et al. Probing p300/CBP associated factor (PCAF)-dependent pathways with a small molecule inhibitor. *ACS Chem. Biol.* **8**, 1311-1323 (2013).

14. Choi, K.C. et al. Epigallocatechin-3-gallate, a histone acetyltransferase inhibitor, inhibits EBV-induced B lymphocyte transformation via suppression of RelA acetylation. *Cancer Res.* **69**, 583-592 (2009).

15. Balasubramanyam, K. et al. Curcumin, a novel p300/CREB-binding protein-specific inhibitor of acetyltransferase, represses the acetylation of histone/nonhistone proteins and histone acetyltransferase-dependent chromatin transcription. *J. Biol. Chem.* **279**, 51163-51171 (2004).

16. Costi, R. et al. Cinnamoyl compounds as simple molecules that inhibit p300 histone acetyltransferase. *J. Med. Chem.* **50**, 1973-1977 (2007).

17. Balasubramanyam, K. et al. Polyisoprenylated benzophenone, garcinol, a natural histone acetyltransferase inhibitor, represses chromatin transcription and alters global gene expression. *J. Biol. Chem.* **279**, 33716-33726 (2004).

18. Balasubramanyam, K., Swaminathan, V., Ranganathan, A. & Kundu, T.K. Small molecule modulators of histone acetyltransferase p300. *J. Biol. Chem.* **278**, 19134-19140 (2003).

19. Ghizzoni, M. et al. 6-alkylsalicylates are selective Tip60 inhibitors and target the acetyl-CoA binding site. *Eur. J. Med. Chem.* **47**, 337-344 (2012).

20. Sorum, A.W. et al. Microfluidic mobility shift profiling of lysine acetyltransferases enables screening and mechanistic analysis of cellular acetylation inhibitors. *ACS Chem. Biol.* **11**, 734-741 (2015).

21. Arif, M. et al. Nitric oxide-mediated histone hyperacetylation in oral cancer: target for a water-soluble HAT inhibitor, CTK7A. *Chem. Biol.* **17**, 903-913 (2010).

22. Hao, J. et al. Selective small molecule targeting β-catenin function discovered by *in vivo* chemical genetic screen. *Cell Rep.* **4**, 898-904 (2013).

23. Sbardella, G. et al. Identification of long chain alkylidenemalonates as novel small molecule modulators of histone acetyltransferases. *Bioorg. Med. Chem. Lett.* **18**, 2788-2792 (2008).

24. Gao, C. et al. Rational design and validation of a Tip60 histone acetyltransferase inhibitor. *Sci. Rep.* **4**, 5372 (2014).

25. Lopes da Rosa, J., Bajaj, V., Spoonamore, J. & Kaufman, P.D. A small molecule inhibitor of fungal histone acetyltransferase Rtt109. *Bioorg. Med. Chem. Lett.* **23**, 2853-2859 (2013).

26. Falk, H. et al. An efficient high-throughput screening method for MYST family acetyltransferases, a new class of epigenetic drug targets. *J. Biomol. Screen.* **16**, 1196-1205 (2011).

27. Lau, O.D. et al. HATs off: selective synthetic inhibitors of the histone acetyltransferases p300 and PCAF. *Mol. Cell.* **5**, 589-595 (2000).

28. Montgomery, D. & Meier, J. Mapping lysine acetyltransferase-ligand interactions by activity-based capture. *Methods Enzymol.* **574**, 105-123 (2016).

29. Seong, A. et al. Delphinidin, a specific inhibitor of histone acetyltransferase, suppresses inflammatory signaling via prevention of NF-κB acetylation in fibroblast-like synoviocyte MH7A cells. *Biochem. Biophys. Res. Commun.* **410**, 581-586 (2011).

30. Ghizzoni, M., Haisma, H. & Dekker, F. Reactivity of isothiazolones and isothiazolone-1-oxides in the inhibition of the PCAF histone acetyltransferase. *Eur. J. Med. Chem.* **44**, 4855-4861 (2009).

31. Thorsheim, K. et al. Disubstituted naphthyl β-D-xylopyranosides: Synthesis, GAG priming, and histone acetyltransferase (HAT) inhibition. *Glycoconj. J.* **33**, 245-257 (2016).

32. Park, W. & Ma, E. Inhibition of PCAF histone acetyltransferase and cytotoxic effect of N-acylanthranilic acids. *Arch. Pharm. Res.* **35**, 1379-1386 (2012).

33. Buczek-Thomas, J., Hsia, E., Rich, C., Foster, J. & Nugent, M. Inhibition of histone acetyltransferase by glycosaminoglycans. *J. Cell. Biochem.* **105**, 108-120 (2008).

34. Furdas, S.D., Shekfeh, S., Kannan, S., Sippl, W. & Jung, M. Rhodanine carboxylic acids as novel inhibitors of histone acetyltransferases. *Med. Chem. Commun.* **3**, 305-311 (2012).

35. Ravindra, K.C., Narayan, V., Lushington, G.H., Peterson, B.R. & Prabhu, K.S. Targeting of histone acetyltransferase p300 by cyclopentenone prostaglandin Δ^{12}-PGJ_2 through covalent binding to Cys^{1438}. *Chem. Res. Toxicol.* **25**, 337-347 (2012).

36. Mai, A. et al. Identification of 4-hydroxyquinolines inhibitors of p300/CBP histone acetyltransferases. *Bioorg. Med. Chem. Lett.* **19**, 1132-1135 (2009).

37. Li, G.-B. et al. Identification of new p300 histone acetyltransferase inhibitors from natural products by a customized virtual screening method. *RCS Adv.* **6**, 61137-61140.

38. Kim, M. et al. Gallic acid, a histone acetyltransferase inhibitor, suppresses β-amyloid neurotoxicity by inhibiting microglial-mediated neuroinflammation. *Mol. Nutr. Food Res.* **55**, 1798-1808 (2011).

39. Lenoci, A. et al. Quinoline-based p300 histone acetyltransferase inhibitors with pro-apoptotic activity in human leukemia U937 cells. *ChemMedChem* **9**, 542-548 (2014).

40. Ornaghi, P., Rotili, D., Sbardella, G., Mai, A. & Filetici, P. A novel Gcn5p inhibitor represses cell growth, gene transcription and histone acetylation in budding yeast. *Biochem. Pharmacol.* **70**, 911-917 (2005).

41. Ruotolo, R. et al. Chemogenomic profiling of the cellular effects associated with histone H3 acetylation impairment by a quinoline-derived compound. *Genomics* **96**, 272-280 (2010).

42. Selvi, R. et al. Inhibition of p300 lysine acetyltransferase activity by luteolin reduces tumor growth in head and neck squamous cell carcinoma (HNSCC) xenograft mouse model. *Oncotarget* **6**, 43806-43818 (2015).

43. Park, S. et al. Selective PCAF inhibitor ameliorates cognitive and behavioral deficits by suppressing NF-κB-mediated neuroinflammation induced by Aβ in a model of Alzheimer's disease. *Int. J. Mol. Med.* **35**, 1109-1118 (2015).

44. Arif, M. et al. Mechanism of p300 specific histone acetyltransferase inhibition by small molecules. *J. Med. Chem.* **52**, 267-277 (2009).

45. Secci, D. et al. Synthesis of a novel series of thiazole-based histone acetyltransferase inhibitors. *Bioorg. Med. Chem.* **22**, 1680-1689 (2014).

46. Sugumar, R., Adithavarman, A., Dakshinamoorthi, A., David, D. & Ragunath, P. Virtual screening of phytochemicals to novel target (HAT) Rtt109 in *Pneumocystis jirovecii* using bioinformatics tools. *J. Clin. Diagn. Res.* **10**, FC05-FC08 (2016).

47. Tohyama, S. et al. Discovery and characterization of NK13650s, naturally occurring p300-selective histone acetyltransferase inhibitors. *J. Org. Chem.* **77**, 9044-9052 (2012).
