# Supplementary material for: Assay interference and off-target liabilities of reported histone acetyltransferase inhibitors
Source: Nat Commun. 2017 Nov 15;8:1527. doi: 10.1038/s41467-017-01657-3 (PMC5688144; doi:10.1038/s41467-017-01657-3)
Supplement: Supplementary file 2 — Descriptions of Additional Supplementary Files [file 41467_2017_1657_MOESM2_ESM.pdf]

Descriptions of Additional Supplementary Files

File Name: Supplementary Dataset 1
Descriptions: File containing SMILES, activity data, and calculated descriptors for reported HAT inhibitors (CSV).

File Name: Supplementary Dataset 2
Descriptions: File containing list of publications and vendors used in the citation and vendor analyses (XLSX).
